# Factors driving adolescent tuberculosis incidence by age and sex in 30 high-tuberculosis burden countries: a mathematical modelling study

**DOI:** 10.1136/bmjgh-2024-015368

**Published:** 2025-03-05

**Authors:** Silvia S Chiang, Megan B Murray, Alexander W Kay, Peter J Dodd

**Affiliations:** 1 Department of Pediatrics, Division of Pediatric Infectious Diseases, Warren Alpert Medical School of Brown University, Providence, Rhode Island, USA; 2 Center for International Health Research, Rhode Island Hospital, Providence, Rhode Island, USA; 3 Department of Global Health and Social Medicine, Harvard Medical School, Boston, Massachusetts, USA; 4 Department of Pediatrics, Baylor College of Medicine, Houston, Texas, USA; 5 Baylor College of Medicine Children’s Foundation-Eswatini, Mbabane, Eswatini; 6 Division of Population Health, The University of Sheffield, Sheffield, UK

**Keywords:** Nutrition, HIV, Tuberculosis

## Abstract

**Introduction:**

During adolescence, tuberculosis incidence rises, with a greater increase in males compared with females. Tuberculosis notifications and estimates infrequently disaggregate adolescent age groups. Moreover, the factors that drive the increases in overall incidence and the male-to-female (MF) ratio remain unclear.

**Methods:**

We constructed a mechanistic model to estimate cumulative *Mycobacterium tuberculosis* infection and tuberculosis disease incidence in the WHO’s 30 high-tuberculosis burden countries (HBCs), which represent 86%–90% of global tuberculosis incidence. We derived infection risk from tuberculosis prevalence and assortative social mixing based on sex and age (10–14 years vs 15–19 years old). We adjusted age subgroup-specific risks of disease progression by age- and sex-specific risks of low body mass index (BMI), pregnancy and postpartum period (PPP) and HIV coinfection. We calculated population attributable fractions (PAFs) to these factors.

**Results:**

In 2019, 91.2 million (95% uncertainty interval (UI) 83.9 to 99.3 million) adolescents in the 30 HBCs had been infected with *M. tuberculosis*, and an estimated 1.0 million (95% UI 0.8 to 1.2 million) developed tuberculosis disease. The median PAF of tuberculosis disease to HIV, modified by antiretroviral therapy, was 1% and highest in Southern Africa. The median PAF for PPP among older adolescents of both sexes was 2.6%. The median PAF to low BMI was 16% and highest in South Asia. The MF risk ratio of tuberculosis disease was 1.2-fold higher among older adolescents, relative to young adolescents. The widening MF risk ratio was attributable mostly to low BMI, with a smaller contribution from sex-assortative social mixing.

**Conclusion:**

Globally, large numbers of adolescents have been infected by *M. tuberculosis* and develop tuberculosis disease. Low BMI is the most important contributor to the overall incidence of tuberculosis disease, as well as to the sex difference that widens with age.

**WHAT IS ALREADY KNOWN ON THIS TOPIC**

- Tuberculosis rates increase during adolescence (10–19 years of age), but the level and drivers of tuberculosis incidence in this age group are uncertain.

**WHAT THIS STUDY ADDS**

- We modelled the contribution to adolescent tuberculosis incidence of low body mass index (BMI), HIV infection, pregnancy and postpartum period and changes in exposure due to social mixing.
- We found a high-tuberculosis incidence during adolescence, with sex differences in tuberculosis rates emerging due to differences in BMI and sex-assortative mixing.

**HOW THIS STUDY MIGHT AFFECT RESEARCH, PRACTICE OR POLICY**

- Nutritional interventions may have an important role in preventing tuberculosis in adolescents, alongside HIV prevention and treatment, and use of tuberculosis preventive therapy.

## Introduction

 Tuberculosis in adolescents, defined by the WHO as people 10–19-years old, substantially impacts global public health.^1^ 90% of the world’s 1.2-billion adolescents live in low- and middle-income countries, where the global tuberculosis burden is concentrated. Until recently, tuberculosis reporting practices grouped young adolescents, or individuals 10- to 14-year olds, with children, and older adolescents, or individuals 15- to 19-year olds, with adults.^2^ In 2020, the WHO requested that countries report disaggregated data for children and adolescents by 5-year age bands, but as of 2022, only 99 of 217 (45.6%) reporting countries have done so.^3^

Tuberculosis disease incidence rises during adolescence. Data from the prechemotherapy era demonstrated that the risk of progression from *Mycobacterium tuberculosis* infection, previously known as ‘latent tuberculosis infection’, to tuberculosis disease peaks in infancy, then decreases to a nadir in the primary school years, and rises again in adolescence.^4^ Global data from the WHO also show that tuberculosis case notifications are much higher among 15–24-year olds than 5–14-year olds.^3^ However, the reasons underlying this risk increase have yet to be identified.

Tuberculosis incidence differs by sex. Adult men have a higher prevalence of bacteriologically confirmed tuberculosis than adult women, with a prevalence ratio of 2.24 (95% CI 1.92 to 2.54).^5^ Globally, tuberculosis notifications in children 0–4 years are slightly higher in males. The sex difference disappears in 5–14-year olds, before reaching a male-to-female (MF) ratio of approximately 1.7 in those over 15 years of age.^6^ One potential explanation for the differences in adults is more use of tobacco and alcohol, which are known risk factors for tuberculosis disease, in men compared with women.^3^ Men and women often have more social interactions with individuals of their own sex,^7^ and this sex-assortative social mixing may amplify tuberculosis incidence among men.^8^

No prior studies have examined the factors that drive adolescent tuberculosis incidence or the differences by sex and age subgroup, that is, young versus older adolescents. During adolescence, social mixing patterns and known tuberculosis risk factors, such as HIV infection, change—sometimes differentially by sex. For example, HIV infection incidence is higher in older adolescents and typically higher in females than males.

To estimate tuberculosis incidence in adolescents and the impact of known risk factors and assortative social mixing, we modelled *M. tuberculosis* infection and progression to tuberculosis disease in the WHO’s 30 high-tuberculosis burden countries (HBCs), which account for 86%–90% of estimated global tuberculosis incidence. We adapted previous work modelling tuberculosis infection to include sex- and age-assortative mixing, and modelled age- and sex-specific changes in risk due to HIV, antiretroviral therapy (ART) coverage, distributions of body mass index (BMI) and pregnancy and postpartum period (PPP). This approach is independent of tuberculosis notification data, meaning that estimates can be compared with notifications.

## Methods

### Overview

**Figure 1:**
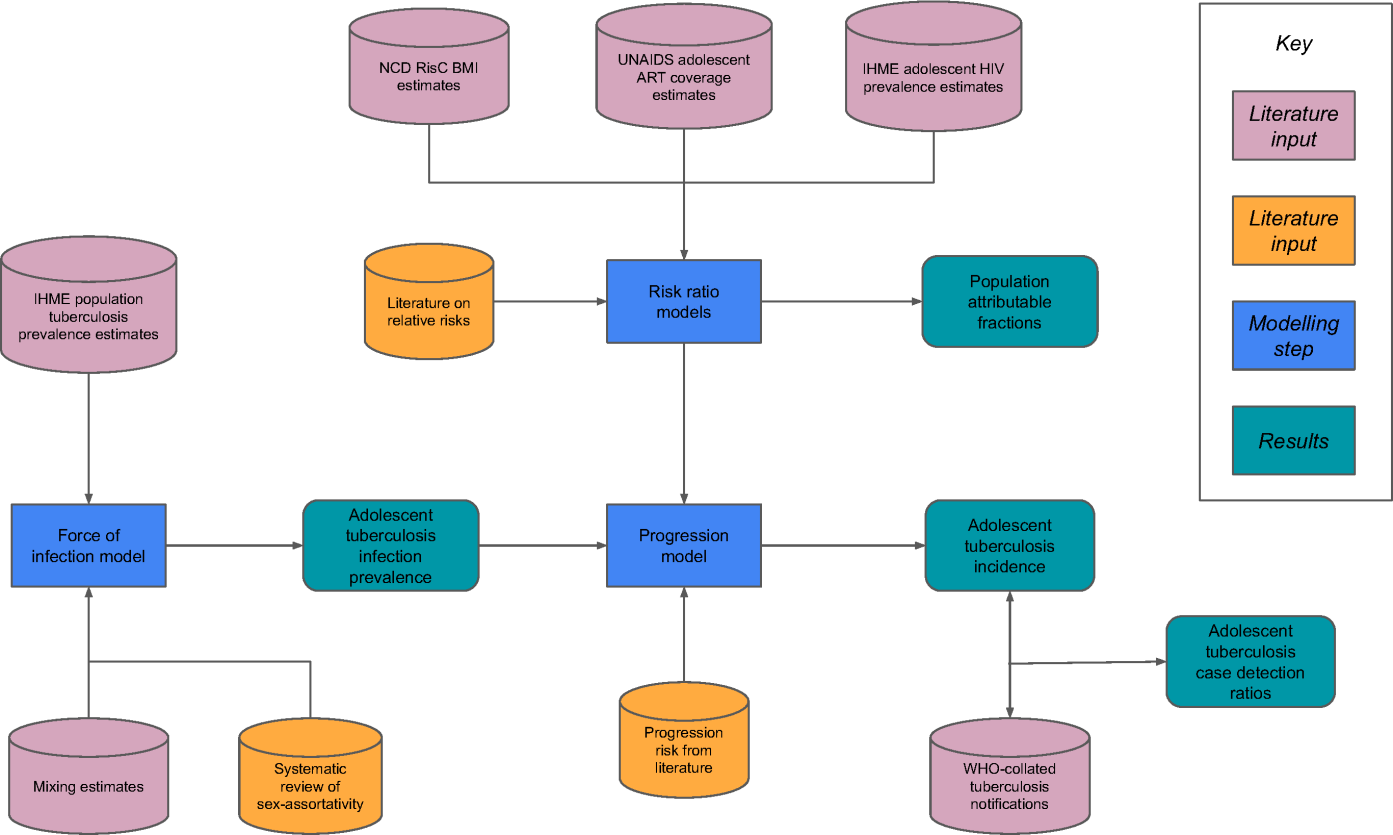
Conceptual summary of data and approach. ART, antiretroviral therapy; BMI, body mass index; IHME, Institute for Health Metrics and Evaluation; UNAIDS, Joint United Nations Programme on HIV/AIDS.

To estimate the 2019 incidence of tuberculosis disease in adolescents in the 30 HBCs, we combined modelled *M. tuberculosis* infection risks with estimates of progression to disease, including the influence of HIV coinfection, with and without ART, and the distribution of BMI, as shown in figure 1. We applied parameters and reported estimates stratified by age subgroup—young adolescents 10–14-years old and older adolescents 15–19-years old—and sex. Recognising that many known tuberculosis risk factors are impacted by gender rather than sex, we nonetheless report sex-disaggregated estimates because the data used in our model were all stratified by sex.

### Risk of tuberculosis infection

Motivated by the need to consider more recent years and revisions in estimated tuberculosis prevalence, we updated the approach of Houben and Dodd,^9^ modifying it in three respects. (1) Because WHO no longer publishes estimates of tuberculosis disease prevalence, in this analysis, we used tuberculosis prevalence estimates from the Institute for Health Metrics and Evaluation (IHME).^10^ (2) We extended the time period for the Gaussian process model of infection risks to 2019. (3) Motivated by evidence that later adolescence may represent a period of higher risk of tuberculosis exposure due to increased social mixing,^11^,^15^ we applied a sex-dependent infection HR to the infection risks experienced by older adolescents compared with young adolescents, for whom we did not consider infection differences by sex.

These age and sex differences in tuberculosis infection risk were derived from the estimates of country-specific age-assortative social mixing from Prem *et al*,^16^ sex assortativity^7^ and the likely patterns of infectious tuberculosis prevalence by age and sex. We assumed that infectious tuberculosis prevalence was distributed by age proportional to per capita tuberculosis notification rates in adults and that its sex ratio matched the WHO estimated MF ratio for tuberculosis incidence. We meta-analysed the resulting HRs for infection rates for older adolescents relative to young adolescents for each WHO region and used predictions for countries lacking the requisite data (online supplemental appendix).

We used this approach to estimate the numbers of female and male adolescents aged 10–14 and 15–19 years in 2019 in each country with cumulative *M. tuberculosis* infection due to primary infection or reinfection within 2 years, accounting for partial protection conferred by previous infection. (We use the term cumulative infection to mean infection at some point resulting in a positive infection test, but not necessarily implying a currently viable infection.) We assumed that assortative mixing is applied to older adolescents and considered random mixing in a sensitivity analysis.

### Progression to tuberculosis disease

Baseline risk of progression to tuberculosis disease within 2 years was taken from a recent individual patient meta-analysis of Martinez *et al*,^17^ which reported risks of 8.8% (95% uncertainty interval (UI) 3.7% to 19.7%) and 10.6% (95% UI 4.4% to 23.3%) for young and older adolescents, respectively. We split this risk between the first and second year following infection as reported by Martinez *et al*^17^ (online supplemental appendix) and applied corresponding risks to those (re)infected within 1 year or 1–2 years. Baseline risk of progression to tuberculosis disease for those infected longer than 2 years ago was taken from Ragonnet *et al*.^18^ Because the slow progression rate of Ragonnet *et al* is for a differently structured model of progression, we also conducted a sensitivity analysis with this rate set to zero.

The risk factors we included were HIV, modified by ART, PPP and undernutrition, captured via the distribution of BMI at each age and sex. We applied these factors as independent incident rate ratios (IRRs) to baseline progression risks and also calculated population attributable fractions (PAFs) for each country and age subgroup. For HIV/ART, we used age- and country-specific estimates of adolescents living with HIV from IHME and estimates of ART coverage from UNAIDS. IRRs were applied based on a systematic review and meta-analysis of the impact of HIV and ART on tuberculosis disease incidence in children.^19^ For PPP, we used UNs estimates of age-specific fertility rates and separate IRRs for PPP based on Zenner *et al*^20^ and Jonsson *et al*’s study.^21^ (online supplemental appendix). For BMI, we used NCD-RisC data for adolescents aged 10–19 years in each country to parametrise an age- and sex-specific BMI distribution.^22^ The BMI distribution was compared with the WHO reference distribution for age and sex,^23^ and a log-linear relationship between BMI and IRR from Lönnroth *et al*^24^ was used to calculate the IRR (online supplemental appendix).

### Estimated incidence of adolescent tuberculosis disease

As our primary outcome, we estimated tuberculosis disease incidence for each age subgroup by applying progression risk, adjusted by age- and country-specific effects of HIV/ART and low BMI, to our corresponding estimated prevalence of cumulative tuberculosis infection, with the effects of social mixing. We conducted sensitivity analyses that did not include the impacts of HIV/ART, BMI and social mixing. To capture uncertainty, all results were calculated within a probabilistic sensitivity analysis combining 200 replicates of all quantities, and reporting medians and 95% quantiles as UIs. We calculated overall and country-specific PAFs to HIV/ART and low BMI.

### Estimated case detection rates (CDRs)

For each country that reported age-disaggregated tuberculosis notifications for adolescents, we calculated the CDR as the per cent of case notifications to our incidence estimate.

### Patient and public involvement

Patients or the public were not involved in the design, or conduct, or reporting or dissemination plans of our research.

All code and data to reproduce this analysis are publicly available at: https://github.com/petedodd/adotb^25^

## Results

### Estimated prevalence of cumulative *M. tuberculosis* infection

We estimated that 91.2 million (95% UI 83.9 to 99.3 million) adolescents in the 30 HBCs—or 11.3% of all adolescents in those countries—had been infected at some point with *M. tuberculosis* infection in 2019. At the country level, the prevalence ranged from 1.8% in Brazil to 26.9% in Lesotho. Of 37.1 million (95% UI 33.8 to 40.5 million) young adolescents with *M. tuberculosis* infection in 2019, 3.0 million (8.0%) were infected less than 1 year prior and 5.9 million (15.6%), less than 2 years prior. Of 53.5 million older adolescents with *M. tuberculosis* infection in 2019, 3.6 million (6.7%) were infected less than 1 year prior and 6.6 million (12.3%), less than 2 years prior (online supplemental appendix).

In many countries, annual risk of infection peaked in the 15–24-year age band (online supplemental appendix). The median risk ratio from social mixing for *M. tuberculosis* infection in older adolescents, relative to young adolescents, was 1.23, ranging from 1.10 in Lesotho to 1.40 in Ethiopia (online supplemental appendix). The impact of social mixing led to an estimated 0.8 million more *M. tuberculosis* infections acquired within the past year in older adolescents, despite the population of young adolescents being larger by 16 million. A sensitivity analysis that did not incorporate social mixing for older adolescents yielded an estimated prevalence of cumulative *M. tuberculosis* infection of 51.7 million (95% UI 47.7 to 56.5 million) or 13.1%, in that age subgroup in the 30 HBCs.

### Population attributable fractions

The median PAF for HIV, modified by ART, was 0.7% in young adolescents (range: 0%–9.6% for individual countries) and 1.4% in older adolescents (range: 0%–16.8%). PAFs for HIV were highest in Lesotho, Mozambique and South Africa. The median PAF for PPP combined, across both sexes, was 0.1% in young adolescents (range: 0%–0.4% for individual countries) and 2.6% in older adolescents (range: 0%–5.5%). The median PAF for PPP combined among females aged 10–19 was 5.2% (range: 0.0%–10.9%, online supplemental appendix). PAFs for PPP were highest in the Central African Republic, Mozambique and Angola. The median PAF for low BMI was 10% in both age subgroups, but varied greatly between countries ranging from −23% for 10–14-year olds in Papua New Guinea to 36% for 10–14-year olds in India. Pakistan and Bangladesh also had PAFs that exceeded 10% for low BMI (online supplemental table 1). Negative PAFs for BMI, as in Papua New Guinea, corresponded to estimated BMI distributions that were higher than the reference distribution.

### Estimated incidence of tuberculosis disease

For our primary outcome, we estimated an incidence of 1.0 million (95% UI 0.8 to 1.2 million) cases among adolescents in the 30 HBCs, of which 0.4 million (95% UI 0.3 to 0.6 million) occurred in young adolescents and 0.6 (95% UI 0.4 to 0.8 million) occurred in older adolescents (table 1). Older adolescents developed tuberculosis disease at a higher rate per capita than young adolescents, with a median risk ratio of 1.5 (range 1.3–1.6; online supplemental appendix). Our sensitivity analysis with no progression ≥2 years after infection reduced the total basecase incidence by 12% to 0.9 million (95% UI 0.7 to 1.2 million).

**Table 1:** Comparison of estimates of total tuberculosis incidence for 30 high-burden countries in 2019

Age	Sex	With risk factors, assortative	With risk factors, random	Without risk factors, assortative	Without risk factors, random	IHME	Snow
10–14 years	all	411 000 (309 000 to 550 000)	411 000 (308 000 to 551 000)	311 000 (240 000 to 400 000)	311 000 (240 000 to 400 000)	241 000 (206 000 to 276 000)	116 000 (40 200 to 193 000)
	female	194 000 (146 000 to 259 000)	194 000 (145 000 to 259 000)	150 000 (116 000 to 192 000)	150 000 (116 000 to 192 000)	140 000 (118 000 to 161 000)	–
	male	218 000 (163 000 to 291 000)	218 000 (163 000 to 291 000)	161 000 (124 000 to 208 000)	161 000 (124 000 to 208 000)	101 000 (87 500 to 115 000)	–
15–19 years	all	615 000 (458 000 to 823 000)	493 000 (374 000 to 648 000)	444 000 (339 000 to 575 000)	358 000 (280 000 to 456 000)	615 000 (458 000 to 823 000)	519 000 (238 000 to 800 000)
	female	266 000 (197 000 to 351 000)	221 000 (168 000 to 288 000)	207 000 (159 000 to 267 000)	172 000 (135 000 to 218 000)	316 000 (259 000 to 373 000)	–
	male	349 000 (259 000 to 467 000)	272 000 (206 000 to 360 000)	238 000 (179 000 to 309 000)	186 000 (145 000 to 238 000)	276 000 (228 000 to 323 000)	–
10–19 years	all	1 030 000 (807 000 to 1 240 000)	904 000 (721 000 to 1 090 000)	755 000 (612 000 to 898 000)	669 000 (550 000 to 788 000)	833 000 (725 000 to 941 000)	635 000 (344 000 to 927 000)
	female	460 000 (348 000 to 617 000)	414 000 (320 000 to 547 000)	356 000 (279 000 to 457 000)	322 000 (256 000 to 408 000)	456 000 (395 000 to 517 000)	–
	male	566 000 (425 000 to 752 000)	490 000 (372 000 to 650 000)	399 000 (311 000 to 510 000)	347 000 (274 000 to 444 000)	377 000 (328 000 to 427 000)	–

Snow = updated results for these countries using the method of Snow *et al*; IHME = Global Burden of Disease results from the Institute of Health Metrics and Evaluation, corresponding to Kyu *et al* and available from https://vizhub.healthdata.org/gbd-results/

IHMEInstitute for Health Metrics and Evaluation

In sensitivity analyses, ignoring the impact of social mixing led to an incidence estimate of 0.9 million (95% UI 0.7 to 1.0 million), with 120 000 fewer cases in older adolescents. Ignoring HIV/ART, low BMI and PPP led to an estimated incidence of 0.8 million (95% UI 0.6 to 0.9 million) and ignoring both social mixing and risk factors, 0.7 million (95% UI 0.6 to 0.8 million) (table 1). The relative ratio of the primary incidence estimate to the sensitivity analysis without social mixing or risk factors was substantially higher in India than in any other country, and much of this difference was due to the consideration of low BMI.

### Estimated CDR

**Figure 2:**
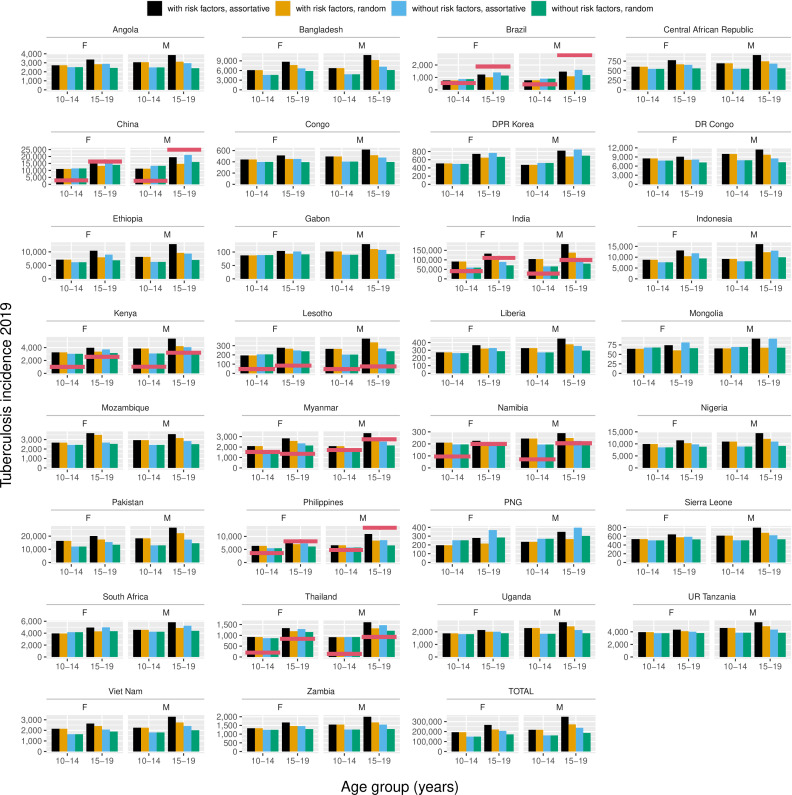
Comparison of estimated tuberculosis incidence for 2019 with and without risk factors and assortative mixing. Red lines denote notified tuberculosis. M, males; F, females.

Disaggregated case notifications for young and older adolescents are available for 2019 from India, China, Philippines, Kenya, Myanmar, Brazil, Thailand, Lesotho and Namibia. The median CDR for young adolescents was 35% (range: 19%–77%), and for older adolescents, 66% (range: 24%–173%). Including assortative social mixing, low BMI and HIV/ART in the model was necessary to estimate an incidence that exceeded notifications in India and Myanmar. In Brazil, China and the Philippines, notifications exceed estimated incidence for at least one age/sex stratification even when including these factors (figure 2).

### MF ratios

**Figure 3:**
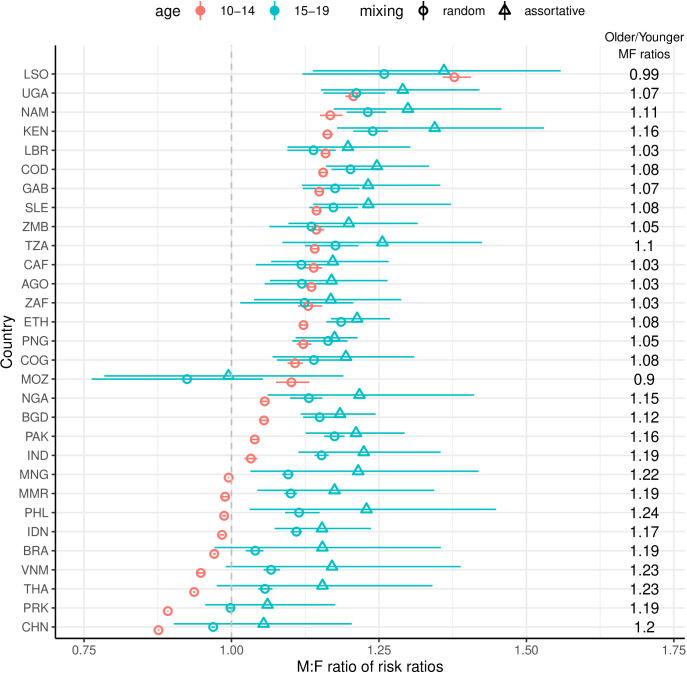
Estimated M:F ratios in tuberculosis incidence for 2019. Points represent means and UIs 95% quantiles. For ages 15–19 years (blue), the triangular points show the influence of sex-assortative mixing on top of sex differences in risk factors. The column of numbers shows the factor change in mean M:F ratio between 10- and 14-year olds and 15- and 19-year olds for assortative mixing. M:F, male:female, MF, male-to-female; UIs, uncertainty intervals.

In almost all countries, the MF ratio of tuberculosis risk from low BMI and HIV/ART increased among older adolescents (figure 3; online supplemental appendix). While in nine countries, the MF ratio was less than 1 for young adolescents, the median value was 1.11. For older adolescents, the basecase MF ratio was greater than 1 in all countries except Mozambique; the median factor increase in the ratio due to sex-assortative mixing in this age subgroup was 1.06. The median factor increase in the MF ratio between the two age subgroups was 1.11.

## Discussion

We have produced estimates of adolescent tuberculosis incidence, accounting for the impact of social mixing on acquisition of *M. tuberculosis* infection and the contributions of low BMI and HIV/ART to progression to tuberculosis disease. According to our model, across the 30 HBCs, 91.2 million adolescents have at some point been infected by *M. tuberculosis* and 1.0 million adolescents developed incident tuberculosis disease in 2019. Low BMI contributed substantially to tuberculosis incidence and drove the discrepancy in incidence between the sexes and its increase with age.

Previous models by Snow *et al*^26^ and the IHME^10^ also estimated adolescent tuberculosis incidence. Neither study analysed patterns by sex or assessed the role of changes in exposure to *M. tuberculosis* or risk factors for progression to disease. For older adolescents, our estimated incidence—when accounting for the impacts of assortative social mixing, low BMI, PPP and HIV/ART—is slightly higher than the estimates by Snow *et al* and the IHME, but within their UIs. However, for young adolescents, our estimated incidence far exceeds those of the other studies, even when we excluded low BMI and HIV/ART from the model. A possible explanation is underdiagnosis of tuberculosis disease in young adolescents, given that Snow *et al* based incidence estimates on notifications, whereas we used a mechanistic model.

Comparing our primary estimates of tuberculosis incidence with notifications in nine countries, we observe median CDRs of 35% in 10–14-year olds and 67% in 15–19-year olds, which compare with global WHO CDR estimates of 49% for 0–14-year olds and 70% for all ages. In some countries and age/sex strata, our primary estimated incidence exceeds notifications, but excluding social mixing and/or risk factors results in a CDR>100%. However, in other countries and age/sex strata, we estimate a CDR>100% under all assumptions, notably for older male adolescents in Brazil, China and the Philippines. Overdiagnosis may play a role in this discrepancy, but more likely, we are underestimating tuberculosis incidence in these strata. One likely explanation is the impact of incarceration, which greatly elevates tuberculosis risk and was not included in our model. Brazil, Russia, China and the Philippines are the countries with the most incident tuberculosis cases among incarcerated individuals in 2019.^27^ In Brazil, individuals 18–24 years of age comprise one-fifths of Brazil’s adult prison population, and more than 21 000 adolescents 12–17-years old were detained in a separate juvenile system.^28^ However, these CDRs reflect all the inputs and assumptions of our approach and should be regarded as hypothesis-generating model checks, rather than definitive statements about programme performance.

A limitation of our approach is its dependence on the estimates of the risk of progression to tuberculosis disease. Although these estimates were derived from a large individual patient data meta-analysis, that study did not stratify progression risk by sex and lacked power to resolve the age dependence of progression within adolescence. Progression risks for the 10–14 and 15–19-year age subgroups had large uncertainty and similar point estimates. This pattern is contrary to expectations of a higher risk in older adolescents based on prechemotherapy literature. Incorporated into our model, the meta-analysis estimates result in higher tuberculosis incidence in young adolescents compared with previous work by Snow *et al* and the IHME, as well as the higher tuberculosis incidence in older adolescents being driven mostly by their greater exposure to *M. tuberculosis*. Finer age resolution in tuberculosis progression estimates would benefit this and other modelling efforts. Another limitation stemming from our use of the progression risks from Martinez *et al*^17^ is that the meta-analysis used data from diverse settings and included unquantified contributions from low BMI and HIV. By using these progression estimates as a baseline and applying IRRs for risk factors, we may have overestimated progression and, therefore, tuberculosis incidence by a uniform factor across settings. True incidence is likely to lie between the estimates we presented with risk factors applied and those based on progression observed in cohort data. We, therefore, presented both estimates separately.

Nonetheless, our PAF estimates are notable and do not depend on progression risks. For most countries, we found that BMI played a more important role in tuberculosis incidence than HIV, but the magnitude of the effect varies hugely by setting. In some settings (eg, Brazil and Papua New Guinea), BMI was higher than reference distributions and, thus, conferred a protective effect. More strikingly, low BMI was implicated in a substantial fraction of tuberculosis incidence in many settings—including over one-thirds of the incidence in India.

While PPP minimally contributed to tuberculosis risk in younger adolescents, the median PAF for PPP in older adolescents was higher than that for HIV. In online supplemental table 1, we report estimates for both sexes combined, and the PAF for pregnancy and postpartum in females is approximately a factor of 2 higher, with a median of over 5% for older female adolescents (online supplemental appendix). PPP, therefore, represents an underappreciated contributor to tuberculosis risk in older female adolescents.

As with PAF estimates, MF incidence ratios do not depend on progression risks. We found that the widening MF ratio in older adolescents was due to lower BMI in males and, to a lesser extent, sex-assortative social mixing (online supplemental appendix). Countries with little or no widening of MF ratios (South Africa, Mozambique and Lesotho) are those where an important role is played by HIV infection, which increases more rapidly with age among female than male adolescents. Similarly, PPP in females counteracts the impact of BMI on sex differences in late adolescence.

While PAFs and MF ratios are robust to limitations in progression estimates, the omission of other risk factors for tuberculosis may have biased our results. We did not include diabetes or tobacco use, but the global prevalence of these conditions in adolescents is <5% and nearly 0%, respectively.^29 30^ Worldwide, approximately, 14% of older adolescents engage in heavy episodic drinking^31^; however, we excluded alcohol use from our model because its relationship with tuberculosis risk is dose dependent,^32^ and current understanding is that the risk increases with chronic exposure.^33^ Although we incorporated BMI, it may not capture the potential effects of micronutrients on disease progression.^34^ Importantly, we assumed that the relationship between BMI and tuberculosis risk did not depend on age. Edwards *et al*^35^ was based on 17–21-year olds and appears compatible with other studies included in Lönnroth *et al*’s study,^24^ but more data are needed to quantify any interaction between age and BMI. Finally, we did not consider tuberculosis preventive therapy (TPT) use, which remains low, including in people living with HIV.^3^

Our findings have important public health implications. Tuberculosis incidence rises during adolescence and, in many settings, peaks in young adulthood. Because they have worse treatment adherence relative to other age groups and tend to congregate in groups, adolescents may play an important role in community transmission of *M. tuberculosis*.^36 37^ In particular, sex-assortative social mixing among older male adolescents contributes to tuberculosis incidence, and male gender has been associated with worse treatment adherence among adolescents.^38^ They are, therefore, a critical target group for early detection and treatment support interventions. By elucidating country-specific PAFs, our study highlights other key targets for intervention and their relative importance in each region. Low BMI is the largest contributor to adolescent tuberculosis incidence globally, and its impact is greatest in South Asia. A recent trial in India demonstrated the effectiveness of nutritional supplementation to reduce tuberculosis incidence among mostly adult household contacts of people with tuberculosis.^39^ Further research is needed to develop and evaluate nutritional interventions for adolescents, who have different nutritional needs than adults and may be more challenging to engage in care. Additionally, continued efforts to prevent HIV transmission in adolescents through expansion of pre-exposure prophylaxis, to promote adherence to ART with the addition of long-acting therapies and to ensure TPT uptake at diagnosis and following tuberculosis exposures, will contribute to decreasing the tuberculosis burden in this age group. Older adolescent females in Southern Africa are a particularly important target group for these tuberculosis/HIV interventions.

In conclusion, we have estimated a substantial burden of cumulative *M. tuberculosis* infection and tuberculosis disease among adolescents worldwide. Low BMI is a major contributor not only to tuberculosis disease incidence—particularly in South Asia—but also to the MF ratio that increases with age.

## supplementary material

10.1136/bmjgh-2024-015368 online supplemental file 1

10.1136/bmjgh-2024-015368 online supplemental table 1

## Data Availability

Data are available in a public, open access repository.
