# Supplementary material for: Factors driving adolescent tuberculosis incidence by age and sex in 30 high-tuberculosis burden countries: a mathematical modelling study
Source: BMJ Glob Health. 2025 Mar 5;10(3):e015368. doi: 10.1136/bmjgh-2024-015368 (PMC11883532; doi:10.1136/bmjgh-2024-015368)
Supplement: online supplemental file 1 [file bmjgh-10-3-s001.pdf]

Factors driving adolescent tuberculosis incidence by age and sex in 30 high tuberculosis burden countries: a mathematical modelling study (Appendix)

Silvia S. Chiang, MD, ScM,[1,2] Megan B. Murray, MD, ScD,[3] Alexander W. Kay, MD,[4] Peter J. Dodd, PhD[5]

1. Department of Pediatrics, Warren Alpert Medical School of Brown University, Providence, Rhode Island, U.S.A.
2. Center for International Health Research, Rhode Island Hospital, 55 Claverick Street, Suite 101, Providence, RI 02906, U.S.A.
3. Department of Global Health and Social Medicine, Harvard Medical School, 641 Huntington Avenue, Boston, MA 02115, U.S.A.
4. Department of Pediatrics, Baylor College of Medicine, 1102 Bates Street, Suite 660, Houston, TX 77030, U.S.A.
5. Baylor College of Medicine Children's Foundation-Eswatini, Cnr Sigwili and Somholo Road, Mbabane, Eswatini, H100
6. Division of Population Health, University of Sheffield, 2004, Regent Court, Sheffield S10 2TN, U.K.

Supplementary methods **2**
- Data sources 2
- Influence of mixing on infection risk 2
- TB infection estimates 5
- Approach to BMI 8
- Approach to HIV 9
- Approach to pregnancy and postpartum 9
- Progression to incident TB disease 10

Supplementary results **11**
- Country-level cumulative TB infection prevalence estimates 11
- Country-level risk ratios from BMI 16
- Country-level TB disease incidence estimates 20
- Country-level PAFs by sex and age 25
- Comparison between methods 31
- Contribution of progression vs infection differences to age differences in TB incidence 32

References **35**

Supplementary methods

Data sources

The following public data sources were used for the inputs for our modelling:

- TB prevalence estimates from IHME: https://vizhub.healthdata.org/gbd-results/
- HIV adolescent prevalence estimates from IHME: https://vizhub.healthdata.org/gbd-results/
- WHO estimates of TB incidence by sex and age: https://www.who.int/teams/global-tuberculosis-programme/data
- United Nation Population Division estimates of population size by sex and age, and age-specific fertility rates: https://population.un.org/wpp/Download/Standard/MostUsed/
- ART coverage estimates from UNAIDS: https://data.unicef.org/topic/hivaids/global-regional-trends/
- BMI estimates in adolescents from NCD RisC: https://ncdrisc.org/data-downloads.html
- Estimated contact matrices: https://github.com/kieshaprem/synthetic-contact-matrices

These were collated for the WHO focus countries, namely:
Angola, Bangladesh, Brazil, Central African Republic, China, Congo, DPR Korea, DR Congo, Ethiopia, Gabon, India, Indonesia, Kenya, Lesotho, Liberia, Mongolia, Mozambique, Myanmar, Namibia, Nigeria, Pakistan, Papua New Guinea, Philippines, Sierra Leone, South Africa, Thailand, Uganda, UR Tanzania, Viet Nam, Zambia

Influence of mixing on infection risk

To consider the potential impact of assortative social mixing on the annual risk of TB infection (ARI) in older adolescents, we followed a 4 step approach:

1. First, WHO estimates of TB incidence by age in 2019 were converted into per capita TB incidence estimates and used to compute per capita TB incidence relative to the 15-24 year old age group (see Figure S1 - each line represents a single country)
2. Secondly, we used the estimated mixing matrices from Prem at al.[1] in each country to construct matrices aggregated over sex and matching our age groups (see Figure S2)
3. Thirdly, these mixing matrices from Step 2 were combined in each country with the relative per capita TB incidence from Step 1 (used as a proxy for relative per capita TB prevalence, ie an assumption of no differences in duration by age and sex), and used to generate implied patterns of ARI by age for each country (see Figure S3). More explicitly, we took

$$RR_{c,a} = \frac{\sum_{a'} M_{c,a,a'} P_{c,a'}}{\sum_{a'} M_{c,1524,a'} P_{c,a'}}$$

where $RR_{c,a}$ is the relative ARI for age group a in country c, $M_{c,a,b}$ is the estimated contact rate

from age group *b* to age group *a* in country *c*, and $P_{c,a}$ is the relative per capita TB prevalence for age group *a* in country *c*.

4. Fourthly, we used a linear interpolation applied to the midpoint of age categories to calculate the relative ARI of 15-19 year olds compared with 10-14 year olds in each country (see Figure S4). This output was then taken forwards into LTBI modelling.

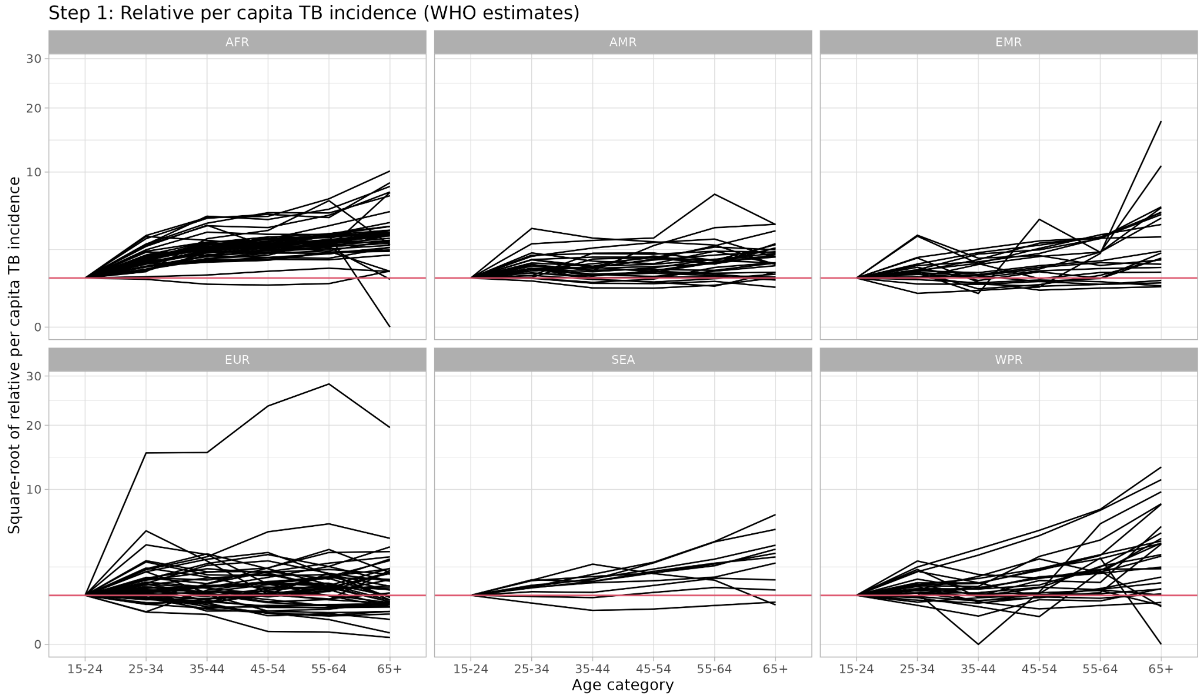

Figure S1 Relative TB per capita incidence by age from WHO estimates. Red line denotes 1

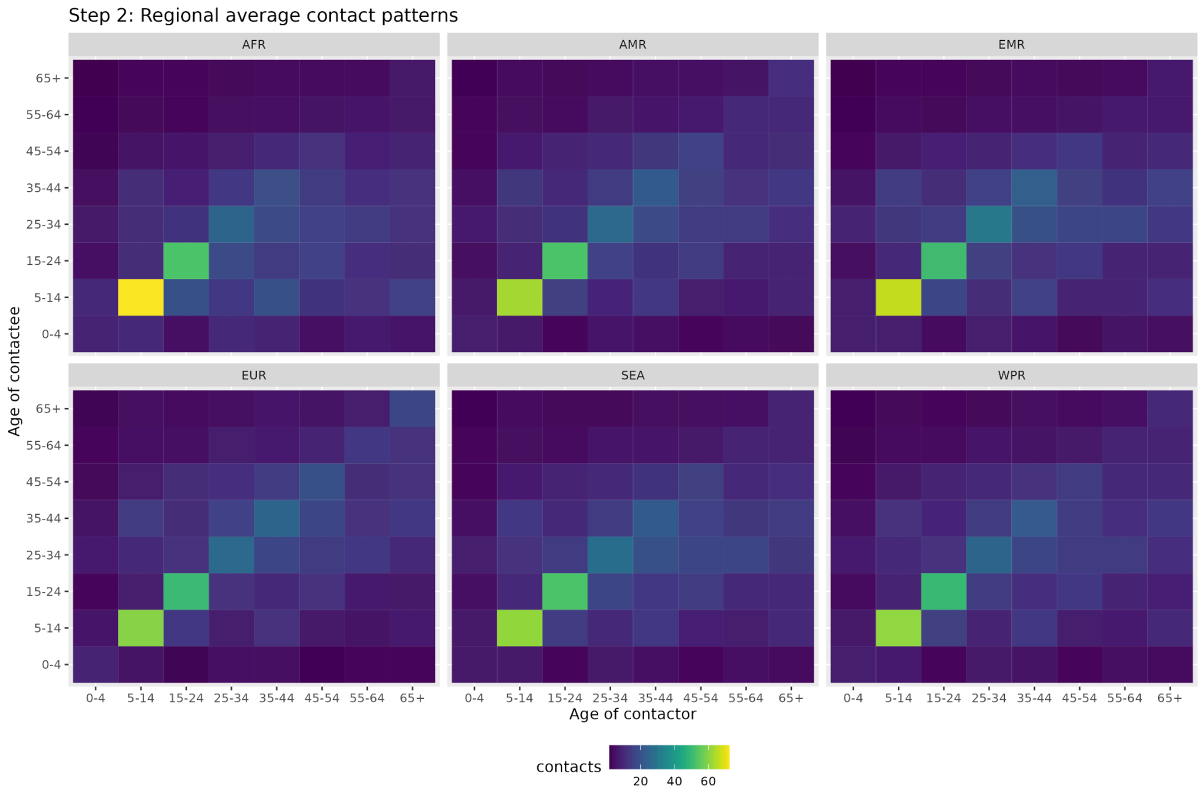

Figure S2 Contact pattern estimates from Prem et al. averaged by WHO region

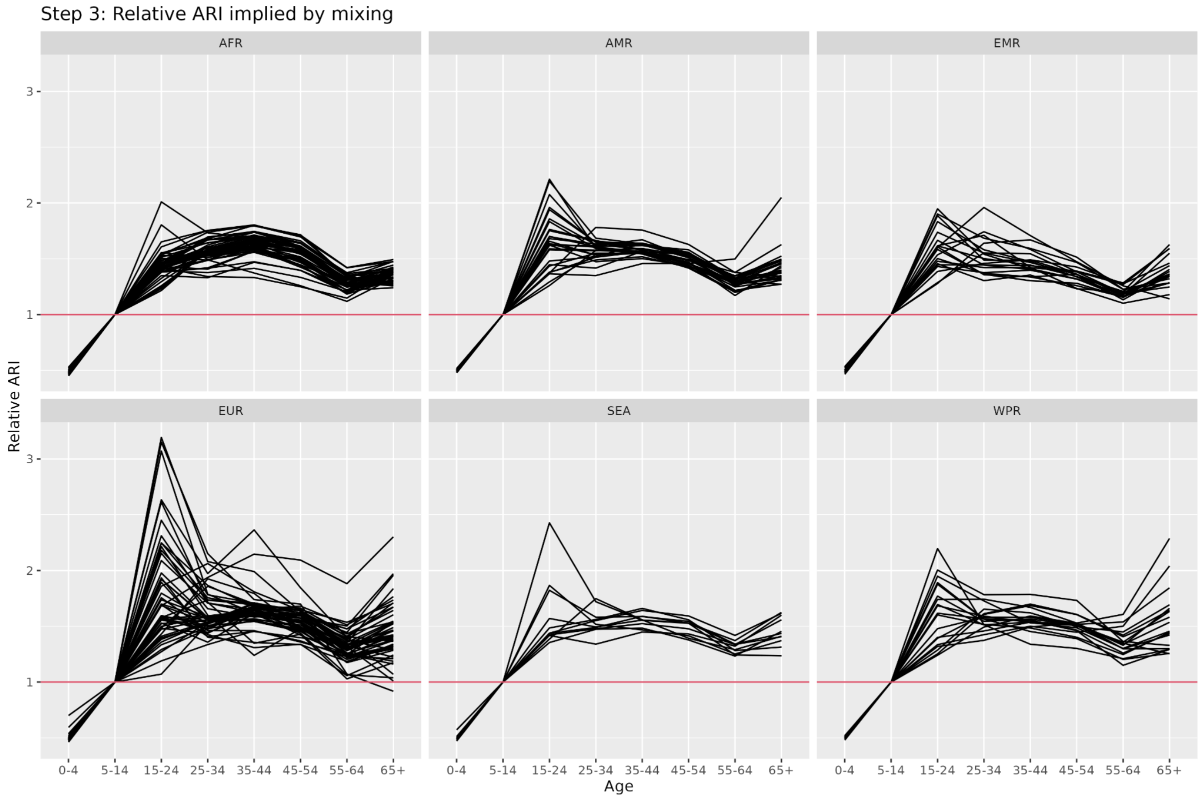

Figure S3 Relative ARI by age implied by Steps 1 & 2 grouped by WHO region

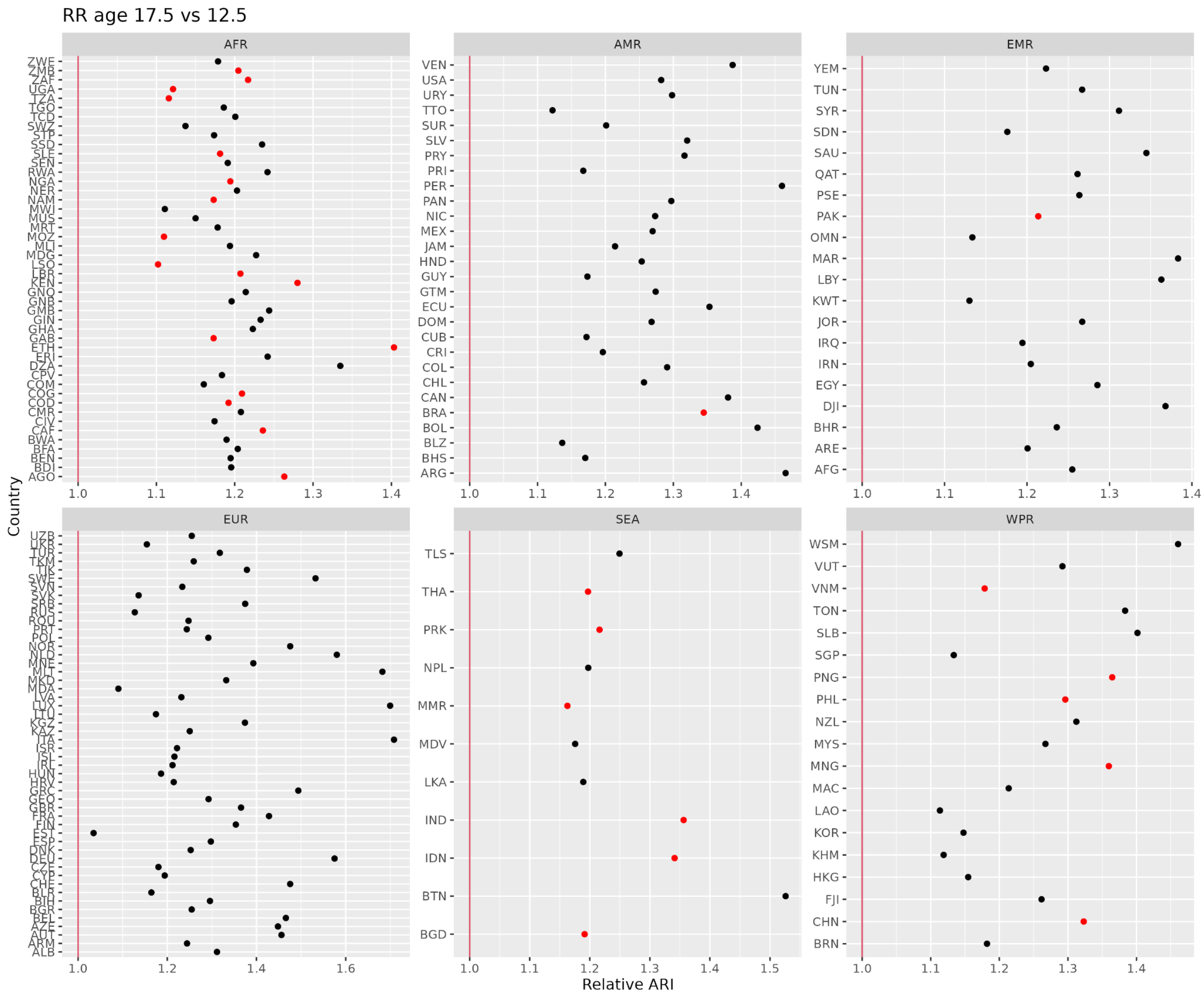

Figure S4 Relative risk for ARI in 15-19 year olds compared to 10-14 year olds (red dots are countries included in our study)

TB infection estimates

To estimate the prevalence of TB infection (TBI), we adapted the previous work of Houben and Dodd[2] in three ways:

1. We used updated estimates of TB disease prevalence from IHME. The rationale for this was threefold: a) we needed to extend beyond the time period for estimated ARI trends considered by the original work; b) new data (especially prevalence surveys) has revised estimates of TB disease in high prevalence countries; c) the switch from WHO prevalence estimates to IHME prevalence estimates was because WHO no longer report estimates of TB prevalence.
2. We included the estimated relative ARI in 15-19 year olds compared with 10-14 year olds (see above) for each country in our base case analysis, and then applied an incremental uncertain factor representing the impact sex-assortative social mixing in those aged 15+ years on exposure to TB infection.
3. We wanted to output TBI prevalence stratified by 3 categories of recency: a) TBI associated with a (re)infection within 1 year; b) TBI associated with a (re)infection ≥ 1 year ago but within 2 years; c) TBI associated with a (re)infection ≥ 1 years ago.

A notable example of updates in burden estimates mentioned in point 1 above is for India. The IHME prevalence estimates for India include the most recent prevalence survey in their uncertainty intervals (see Figure S5). However, these prevalence estimates have a different trend and level from the 2014 WHO estimates used as inputs in Houben and Dodd[2] (see Figure S6)

To account for the increased exposure to TB infection in males aged 15+ years due to their increased social mixing with males (who typically have higher TB prevalence), we combined estimates of TB sex ratio and review data on sex-assortativity. For each country, we calculated a sex ratio in TB prevalence among those aged 15+ years, *sr,* using the ratio of WHO-estimated TB incidence in those aged 15+ years. The review of social contact surveys by Horton et al. found that typically 56% (IQR 54%–58%) of male contacts were with males, and 59% (IQR 57%–63%) of female contacts were with female. We summarized this as 56.5% (IQR: 54%-64%) of contacts being with the same sex, and represented this parameter α as distributed according to a beta distribution B(30.67915,23.62023). We then applied additional factors F_m and F_f to male and female infection rates, respectively, where:

$$F_m = 2\left(\frac{\alpha}{1+sr} + \frac{1-\alpha}{1+1/sr}\right) \qquad F_f = 2\left(\frac{1-\alpha}{1+sr} + \frac{\alpha}{1+1/sr}\right)$$

Other than these changes, the methods are as reported in Houben and Dodd.[2]

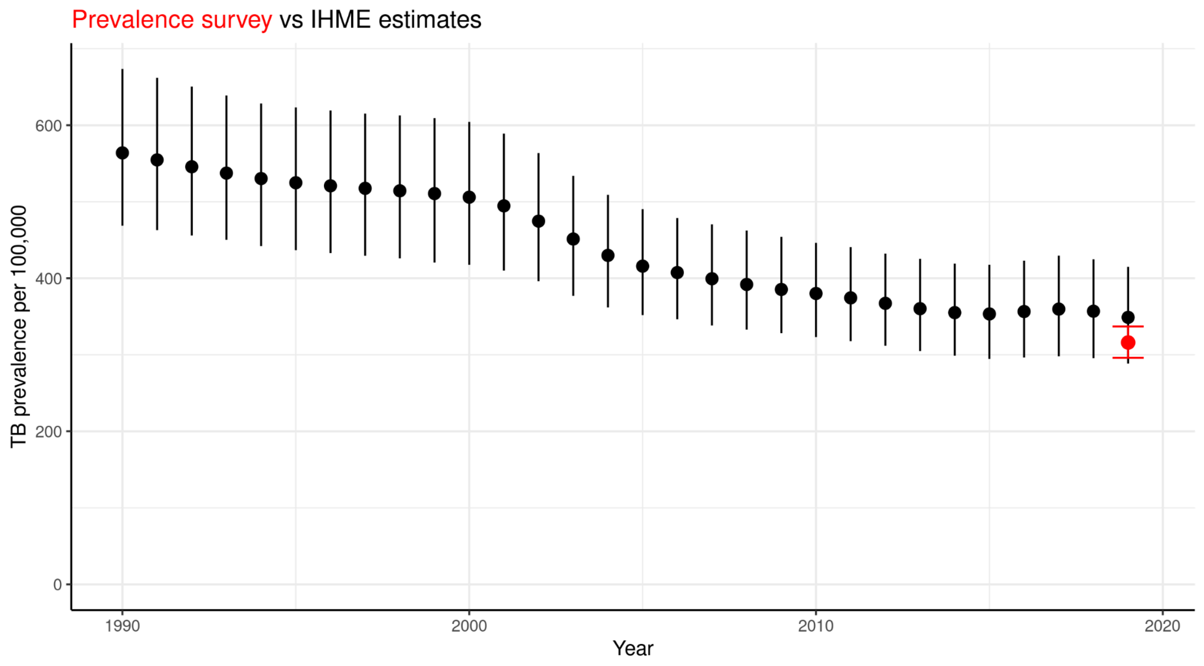

Figure S5 Per capita TB prevalence for India

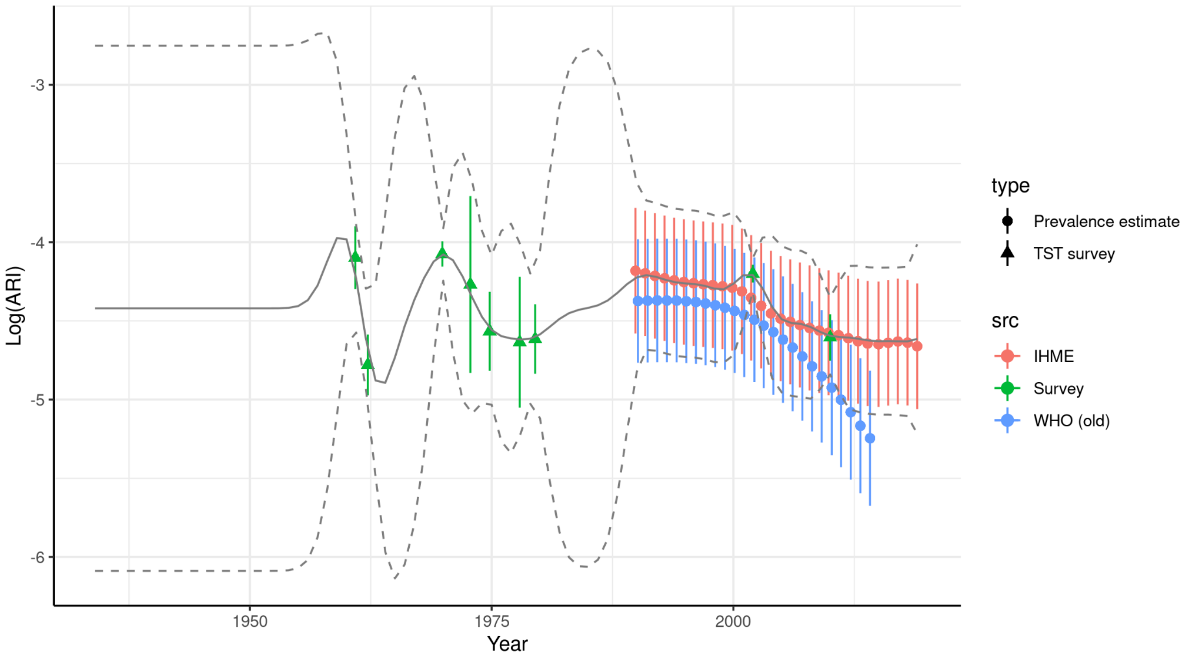

Figure S6 Comparison of the Gaussian process model of log(ARI) for India with original data in Houben and Dodd[2]

Approach to BMI

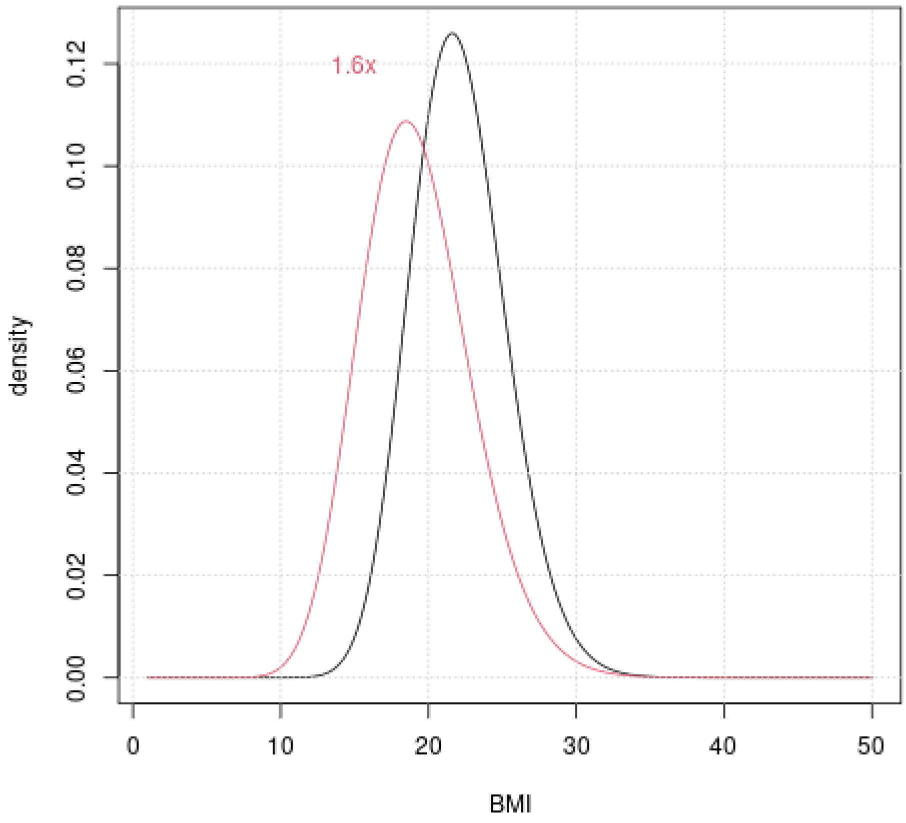

Figure S7 Example of approach to BMI for females aged 19 years in India.
Red is fit to country-specific NCD-RisC estimates; black is fit to WHO reference distribution for this age/sex. The population mean incident rate ratio implied by this shift in BMI distribution and the relationship from Lönnroth et al.[3] is 1.6

Previous work has quantified the incidence risk ratio associated with a given body mass index (BMI).[3] We used the estimate of Lönnroth et al.[3] of a 13.8 (95% CI 13.4−14.2) % factor increase per unit BMI decrease to parametrize an uncertain parameter, with mean t=log(1-0.138) slope of the log incidence rate ratio with respect to BMI.

In order to estimate the change in TB disease incidence risk due to a particular prevalence of thinness in a population, we needed to model the change in the population BMI distribution corresponding to a given prevalence of thinness.

For each country, single-year age group, and sex, we fitted a gamma distribution for the population BMI to NCD-RisC estimates. We used data for 2016 because they included estimates of the mean BMI as well as the +2SD, +1SD, -1SD, and -2SD BMI reference values for each country/sex/age. The estimates of mean BMI differed by an average of <1% between 2016 and 2019. We chose gamma distribution parameters that numerically minimized the sum-of-squares difference between the 5 estimated reference BMI values above and those of the distribution. There were convergence problems for 7 country-ages for females - here average parameters were substituted. We also fitted

gamma distributions to the WHO reference populations for each sex and age from: https://www.who.int/tools/growth-reference-data-for-5to19-years/indicators/bmi-for-age

The moment generating function of the gamma distribution with parameters θ and k is $E[e^{tX}] = 1/(1-t\theta)^k$, which allows us to compute analytically the unnormalized mean of a risk of an exponentially increasing risk in a gamma-distributed population BMI. Finally, the risk ratio for a given population is calculated as $RR= (1-t\theta)^k/(1-t\theta^*)^{k^*}$, where k^* and θ^* are the gamma parameters for the WHO reference distribution at the same age and sex. We propagated the uncertainty SD for t calculated from the 95% confidence intervals to calculate an SD in each RR. Uncertainties in these RRs were propagated using the delta method. An illustrative example of our calculation for a single country, year and sex is shown in Figure S7. Our estimates of TB disease incidence risk ratio due to BMI in each country is reported in Table S4. Figure S9 shows trends in RRs due to BMI by single year age group.

Approach to HIV

To characterize risk factors in each country, we used IHME estimates of HIV prevalence by sex and five-year age group in each country, and UN estimates of numbers in these sex and age groups receiving ART. We modelled the incidence rate ratio due to HIV as

$$IRR_{HIV} = R_{hiv} \times (1 - art + art \times R_{art})$$

where art is the ART coverage in each group, R_{hiv} is the IRR for untreated HIV following a log-normal distribution LN(2.066863,0.2800718), and R_{art} is the factor reduction in TB risk among PLHIV due to ART, following a log-normal distribution LN(-1.203973,0.150482). These are based on a systematic review and meta-analysis in children and young adolescents.[4] Samples from these distributions were applied to HIV prevalences sampled from gamma distributions fitted to match the mean and standard deviation of estimates for each country/sex/age-group.

Approach to pregnancy and postpartum

To capture the impact of pregnancy and postpartum on TB incidence in females, we used UN World Population Prospects 2024 estimates of age specific fertility rates (ASFR) for 2019 by country and 5-year age group to estimate the prevalence of pregnancy (assuming 9 months duration) and postpartum (defined as 6 months duration). To quantify the increased risk during pregnancy and postpartum we used estimates from electronic healthcare record-based cohorts from Zenner et al.[5] and Jonsson et al.[6] We pooled these estimates using inverse variance-weighting to arrive at an incidence rate ratio during pregnancy of 1.39 (1.11 to 1.65) modelled as LN(0.3208441,0.09876595), and an IRR during the 6 month postpartum period of 1.91 (1.47 to 2.35) modelled as LN(0.6478868,0.115937).

Progression to incident TB disease

We based our risk of progression to TB disease following recent infection on age group-specific estimates from the individual patient meta-analysis of Martinez et al.[7] , see Table S1. To differentiate between risks of progression within 1 year and between 1 and 2 years following infection, we used the reported results that 89% of progression was within 1 year and 98% within 2 years to scale the reported 2 year progression probabilities (i.e. 8.8% (3.7-19.7)% for 10-14 year olds and 10.6% (4.4-23.3)% for 14-19 year olds) to <1 year progression probabilities (see Table S1), by applying a factor f=89/98 to the mean and $\sqrt{f}$ to the uncertainty widths, and progression probabilities for ≥1 year and <2 years by applying factor $1 - f$ to the mean and $\sqrt{1 - f}$ to the uncertainty widths (see Table S1).

Table S1 Progression parameters by age for <1 year, 1 to <2 years, and ≥ 2 years after infection
LN = log-normal distribution, B=beta distribution

**Parameter**	**Distribution**	**Mean (95% uncertainty)**	**Source**
Probability of TB ≤ 1 years of infection, 10-14 years	B(3.62, 41.69)	8.0% (2.1-17.4)%	Martinez et al.[7]
Probability of TB ≤ 1 years of infection, 15-19 years	B(3.68,34.59)	9.6% (2.6-20.6)%	Martinez et al.[7]
Probability of TB ≥1 & <2 years of infection, 10-14 years	LN(-4.81,1.10)	1.5% (0.1-6.8)%	Martinez et al.[7]
Probability of TB ≥1 & <2 years of infection, 15-19 years	LN(-4.63,1.09)	1.8% (0.1-8.3)%	Martinez et al.[7]
Rate of progression ≥ 2 years after infection	LN(-6.89,0.58)	0.12% (0.03-3.16)%	Ragonnet et al.[8]

Supplementary results

Country-level cumulative TB infection prevalence estimates

Table S2 Cumulative TB infection prevalence by country

ISO3	Age 10-14 years			Age 15-19 years		
	**All TBI**	**Acquired <2 years ago**	**Acquired <1 year ago**	**All TBI**	**Acquired <2 years ago**	**Acquired <1 year ago**
AGO	636 000 (511 000 to 758 000)	91 300 (56 700 to 142 000)	46 100 (24 600 to 76 600)	764 000 (652 000 to 881 000)	85 500 (52 900 to 131 000)	44 000 (23 700 to 73 900)
BGD	1 260 000 (1 070 000 to 1 530 000)	170 000 (97 400 to 257 000)	88 000 (48 700 to 155 000)	2 010 000 (1 740 000 to 2 330 000)	197 000 (112 000 to 301 000)	104 000 (57 600 to 182 000)
BRA	207 000 (167 000 to 251 000)	31 700 (18 400 to 46 600)	16 000 (7 800 to 26 600)	351 000 (292 000 to 415 000)	44 900 (26 100 to 66 300)	23 200 (11 400 to 38 700)
CAF	120 000 (96 300 to 144 000)	21 100 (12 100 to 34 300)	10 500 (5 380 to 18 900)	150 000 (126 000 to 176 000)	21 000 (12 100 to 33 800)	10 600 (5 460 to 19 200)
CHN	2 830 000 (2 180 000 to 3 460 000)	436 000 (271 000 to 666 000)	230 000 (128 000 to 378 000)	4 400 000 (3 600 000 to 5 200 000)	551 000 (342 000 to 841 000)	297 000 (165 000 to 488 000)
COD	1 900 000 (1 620 000 to 2 240 000)	288 000 (177 000 to 446 000)	144 000 (75 800 to 237 000)	2 230 000 (1 950 000 to 2 600 000)	258 000 (155 000 to 401 000)	131 000 (70 400 to 216 000)
COG	97 100 (84 500 to 113 000)	14 700 (10 700 to 20 100)	7 440 (4 750 to 10 600)	118 000 (105 000 to 131 000)	13 700 (9 950 to 18 600)	7 050 (4 490 to 10 100)

ETH	1 520 000 (1 220 000 to 1 830 000)	225 000 (138 000 to 355 000)	115 000 (62 500 to 195 000)	2 300 000 (1 950 000 to 2 680 000)	271 000 (166 000 to 426 000)	142 000 (75 800 to 241 000)
GAB	21 400 (17 700 to 25 700)	3 310 (1 980 to 5 490)	1 670 (913 to 2 950)	26 900 (23 100 to 31 300)	3 150 (1 890 to 5 200)	1 610 (874 to 2 830)
IDN	1 830 000 (1 510 000 to 2 230 000)	276 000 (161 000 to 422 000)	147 000 (80 300 to 241 000)	2 900 000 (2 430 000 to 3 480 000)	354 000 (205 000 to 544 000)	192 000 (106 000 to 317 000)
IND	14 500 000 (11 300 000 to 17 600 000)	2 310 000 (1 390 000 to 3 490 000)	1 180 000 (629 000 to 1 920 000)	22 200 000 (18 400 000 to 26 400 000)	2 870 000 (1 720 000 to 4 360 000)	1 510 000 (798 000 to 2 440 000)
KEN	708 000 (574 000 to 867 000)	116 000 (69 300 to 183 000)	58 200 (29 600 to 102 000)	933 000 (809 000 to 1 100 000)	123 000 (72 500 to 194 000)	63 000 (32 300 to 110 000)
LBR	65 500 (52 200 to 81 900)	9 840 (6 070 to 14 400)	5 030 (2 830 to 8 100)	86 000 (70 500 to 103 000)	9 780 (6 060 to 14 200)	5 080 (2 880 to 8 200)
LSO	48 600 (39 200 to 60 000)	7 480 (4 500 to 11 800)	3 720 (2 090 to 6 310)	67 200 (56 300 to 80 100)	7 650 (4 630 to 12 100)	3 840 (2 160 to 6 490)
MMR	403 000 (326 000 to 480 000)	62 700 (36 800 to 102 000)	31 900 (14 800 to 53 300)	625 000 (528 000 to 742 000)	71 600 (41 800 to 117 000)	36 900 (17 100 to 61 200)
MNG	17 100 (13 400 to 21 200)	2 530 (1 490 to 3 880)	1 270 (669 to 2 040)	21 400 (17 500 to 25 400)	2 630 (1 540 to 4 010)	1 360 (714 to 2 180)
MOZ	588 000 (476 000 to 725 000)	91 100 (54 700 to 141 000)	45 500 (24 600 to 78 400)	713 000 (586 000 to 848 000)	82 100 (49 600 to 127 000)	41 400 (22 600 to 71 500)
NAM	51 000 (40 900 to 63 000)	6 890 (4 160 to 10 300)	3 440 (1 940 to 5 540)	66 600 (55 700 to 79 000)	6 500 (4 030 to 9 800)	3 300 (1 860 to 5 260)
NGA	2 160 000 (1 720 000 to 2 650 000)	317 000 (183 000 to 464 000)	161 000 (79 100 to 268 000)	2 840 000 (2 410 000 to 3 340 000)	302 000 (174 000 to 443 000)	156 000 (77 000 to 261 000)
PAK	3 120 000 (2 560 000 to 3 870 000)	468 000 (268 000 to 757 000)	234 000 (114 000 to 416 000)	4 450 000 (3 780 000 to 5 220 000)	493 000 (283 000 to 800 000)	251 000 (124 000 to 442 000)

PHL	1 170 000 (967 000 to 1 410 000)	208 000 (137 000 to 320 000)	104 000 (56 800 to 171 000)	1 600 000 (1 370 000 to 1 900 000)	244 000 (160 000 to 375 000)	126 000 (69 000 to 206 000)
PNG	59 400 (49 500 to 71 500)	9 700 (6 430 to 14 800)	4 920 (2 780 to 7 880)	82 600 (70 500 to 98 800)	11 600 (7 720 to 17 700)	6 060 (3 410 to 9 680)
PRK	118 000 (94 800 to 144 000)	18 700 (11 800 to 29 000)	9 470 (5 140 to 15 700)	190 000 (159 000 to 221 000)	24 100 (15 300 to 37 700)	12 500 (6 780 to 20 700)
SLE	133 000 (108 000 to 165 000)	18 900 (12 300 to 30 200)	9 480 (5 460 to 16 700)	171 000 (143 000 to 203 000)	18 100 (11 900 to 28 600)	9 250 (5 290 to 16 500)
THA	190 000 (157 000 to 228 000)	33 900 (19 000 to 53 600)	17 300 (8 690 to 29 900)	300 000 (246 000 to 350 000)	42 100 (23 500 to 66 200)	21 900 (11 000 to 37 900)
TZA	899 000 (738 000 to 1 160 000)	139 000 (85 300 to 223 000)	70 200 (38 200 to 124 000)	1 080 000 (907 000 to 1 300 000)	122 000 (74 100 to 196 000)	62 200 (33 900 to 109 000)
UGA	420 000 (346 000 to 509 000)	67 700 (40 700 to 105 000)	34 300 (18 300 to 57 900)	519 000 (442 000 to 612 000)	61 700 (37 200 to 95 000)	31 600 (16 800 to 53 000)
VNM	402 000 (330 000 to 483 000)	63 700 (41 100 to 96 900)	32 300 (17 300 to 54 800)	571 000 (479 000 to 674 000)	67 100 (43 200 to 103 000)	34 500 (18 600 to 58 600)
ZAF	1 020 000 (827 000 to 1 210 000)	153 000 (90 200 to 230 000)	76 900 (40 500 to 126 000)	1 320 000 (1 120 000 to 1 510 000)	153 000 (91 200 to 233 000)	78 200 (40 400 to 129 000)
ZMB	306 000 (253 000 to 366 000)	46 100 (30 000 to 76 100)	23 500 (13 100 to 44 300)	393 000 (333 000 to 455 000)	44 700 (29 100 to 74 500)	23 200 (12 900 to 43 200)
TOTAL	36 800 000 (33 500 000 to 40 300 000)	5 710 000 (4 680 000 to 6 920 000)	2 910 000 (2 290 000 to 3 840 000)	53 500 000 (49 400 000 to 58 400 000)	6 560 000 (5 300 000 to 8 080 000)	3 430 000 (2 640 000 to 4 550 000)

Table S3 Total prevalence of cumulative TB infection by sex and age, influenced by demography and mixing assumptions

sex	acat	mixing	TB infection with 1 year	TB infection 1-2 years ago	All TB infection
F	10-14	assortative	1 400 000 (1 110 000 to 1 840 000)	2 750 000 (2 260 000 to 3 330 000)	17 700 000 (16 200 000 to 19 400 000)
M	10-14	assortative	1 510 000 (1 180 000 to 2 000 000)	2 960 000 (2 420 000 to 3 590 000)	19 100 000 (17 400 000 to 20 900 000)
F	15-19	assortative	1 590 000 (1 210 000 to 2 120 000)	3 040 000 (2 500 000 to 3 750 000)	25 500 000 (23 600 000 to 27 900 000)
M	15-19	assortative	1 840 000 (1 400 000 to 2 460 000)	3 510 000 (2 800 000 to 4 340 000)	27 900 000 (25 800 000 to 30 600 000)
F	10-14	random	1 400 000 (1 110 000 to 1 850 000)	2 750 000 (2 260 000 to 3 320 000)	17 700 000 (16 200 000 to 19 400 000)
M	10-14	random	1 510 000 (1 180 000 to 1 990 000)	2 960 000 (2 420 000 to 3 580 000)	19 100 000 (17 400 000 to 20 900 000)
F	15-19	random	1 280 000 (997 000 to 1 680 000)	2 500 000 (2 050 000 to 3 040 000)	24 600 000 (22 700 000 to 26 900 000)
M	15-19	random	1 380 000 (1 070 000 to 1 830 000)	2 700 000 (2 200 000 to 3 320 000)	26 700 000 (24 500 000 to 29 200 000)

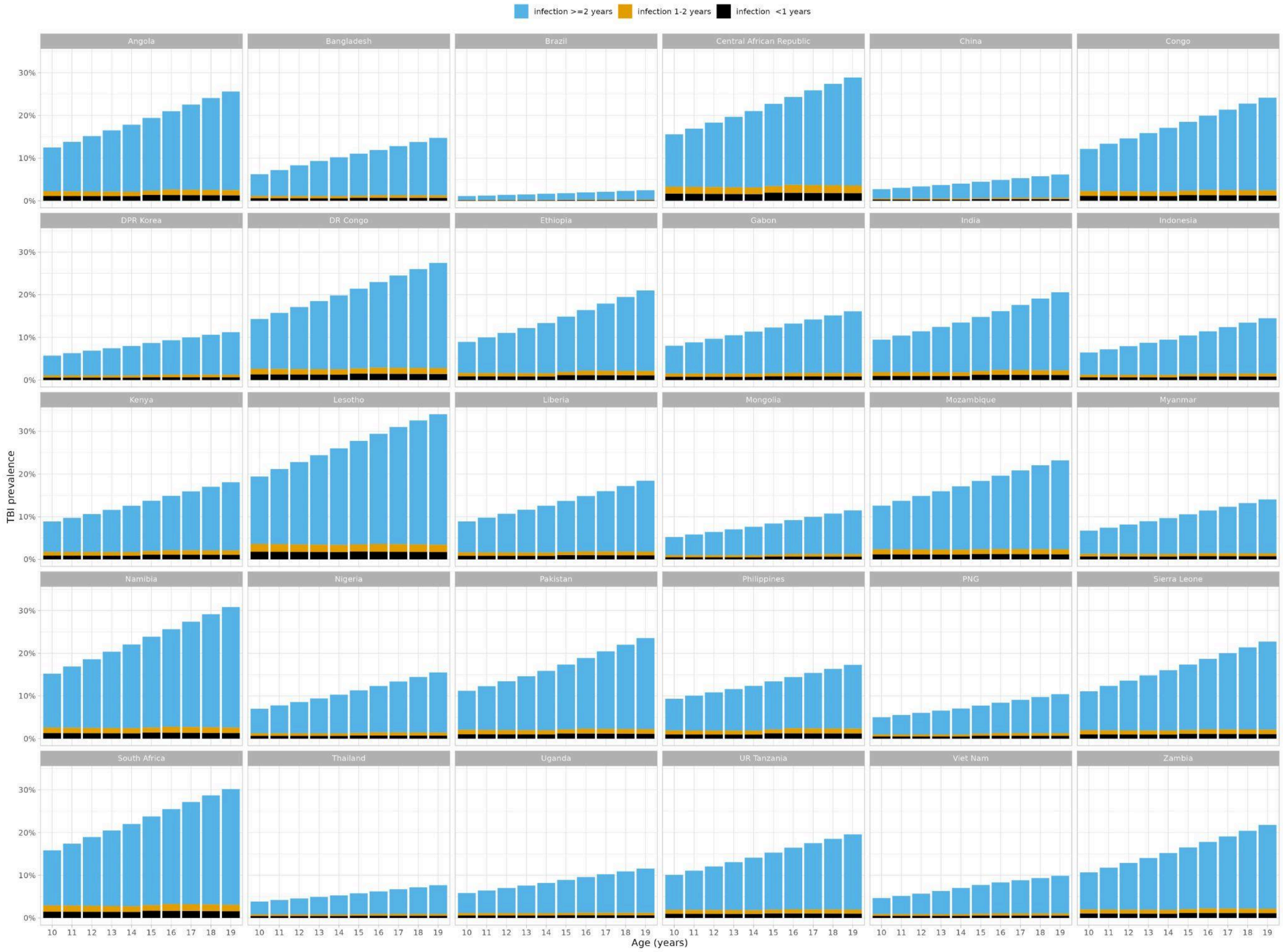

Figure S8 Cumulative TB infection prevalence by country and single-year age group

Country-level risk ratios from BMI

Table S4 Estimates of risk ratios for TB due to BMI in adolescents aged 10-14 and 15-19 by sex and country

Country	Females, 10-14	Females, 15-19	Males, 10-14	Males, 15-19
Angola	1.06 (1.06 to 1.07)	1.00 (1.00 to 1.00)	1.22 (1.21 to 1.22)	1.29 (1.28 to 1.30)
Bangladesh	1.32 (1.31 to 1.33)	1.24 (1.23 to 1.24)	1.40 (1.38 to 1.41)	1.51 (1.48 to 1.53)
Brazil	0.88 (0.88 to 0.88)	0.84 (0.84 to 0.85)	0.85 (0.85 to 0.86)	0.91 (0.91 to 0.91)
Central African Republic	1.06 (1.06 to 1.07)	1.00 (1.00 to 1.01)	1.22 (1.21 to 1.23)	1.30 (1.29 to 1.31)
China	0.96 (0.96 to 0.96)	0.94 (0.94 to 0.95)	0.84 (0.84 to 0.85)	0.92 (0.92 to 0.92)
Congo	1.07 (1.07 to 1.07)	0.99 (0.99 to 0.99)	1.19 (1.18 to 1.19)	1.27 (1.26 to 1.28)
DPR Korea	1.02 (1.02 to 1.02)	0.97 (0.97 to 0.97)	0.91 (0.91 to 0.91)	0.97 (0.97 to 0.97)
DR Congo	1.08 (1.08 to 1.09)	1.02 (1.02 to 1.03)	1.25 (1.24 to 1.26)	1.34 (1.33 to 1.35)
Ethiopia	1.14 (1.13 to 1.14)	1.08 (1.08 to 1.09)	1.28 (1.27 to 1.29)	1.36 (1.35 to 1.37)
Gabon	0.97 (0.97 to 0.97)	0.91 (0.91 to 0.91)	1.12 (1.12 to 1.13)	1.19 (1.19 to 1.20)
India	1.54 (1.52 to 1.56)	1.47 (1.46 to 1.49)	1.59 (1.56 to 1.62)	1.72 (1.69 to 1.75)
Indonesia	1.15 (1.14 to 1.16)	1.08 (1.08 to 1.08)	1.13 (1.13 to 1.14)	1.23 (1.22 to 1.24)

Kenya	1.04 (1.04 to 1.04)	0.97 (0.97 to 0.97)	1.21 (1.20 to 1.22)	1.28 (1.28 to 1.29)
Lesotho	0.87 (0.86 to 0.87)	0.81 (0.80 to 0.82)	1.19 (1.19 to 1.20)	1.26 (1.25 to 1.27)
Liberia	1.03 (1.03 to 1.03)	1.00 (1.00 to 1.00)	1.19 (1.19 to 1.20)	1.26 (1.25 to 1.27)
Mongolia	0.95 (0.95 to 0.95)	0.89 (0.89 to 0.90)	0.95 (0.95 to 0.95)	1.00 (1.00 to 1.00)
Mozambique	0.98 (0.98 to 0.98)	0.93 (0.93 to 0.93)	1.09 (1.09 to 1.09)	1.14 (1.13 to 1.14)
Myanmar	1.24 (1.23 to 1.24)	1.17 (1.16 to 1.18)	1.22 (1.21 to 1.23)	1.32 (1.31 to 1.33)
Namibia	1.01 (1.01 to 1.01)	0.95 (0.95 to 0.95)	1.18 (1.17 to 1.18)	1.26 (1.25 to 1.26)
Nigeria	1.16 (1.15 to 1.16)	1.08 (1.08 to 1.08)	1.23 (1.22 to 1.23)	1.32 (1.31 to 1.33)
PNG	0.77 (0.77 to 0.78)	0.71 (0.70 to 0.72)	0.87 (0.86 to 0.87)	0.88 (0.87 to 0.88)
Pakistan	1.35 (1.34 to 1.37)	1.25 (1.24 to 1.26)	1.41 (1.39 to 1.43)	1.52 (1.50 to 1.54)
Philippines	1.16 (1.16 to 1.17)	1.10 (1.10 to 1.10)	1.15 (1.14 to 1.16)	1.24 (1.23 to 1.25)
Sierra Leone	1.04 (1.04 to 1.05)	0.99 (0.98 to 0.99)	1.20 (1.19 to 1.21)	1.27 (1.26 to 1.28)
South Africa	0.88 (0.87 to 0.88)	0.74 (0.74 to 0.75)	0.98 (0.98 to 0.99)	1.02 (1.01 to 1.02)
Thailand	1.06 (1.06 to 1.07)	1.00 (1.00 to 1.00)	1.00 (0.99 to 1.00)	1.08 (1.07 to 1.08)
UR Tanzania	1.03 (1.02 to 1.03)	0.96 (0.96 to 0.96)	1.17 (1.16 to 1.18)	1.24 (1.23 to 1.25)
Uganda	0.99 (0.99 to 0.99)	0.93 (0.92 to 0.93)	1.20 (1.19 to 1.21)	1.26 (1.25 to 1.27)

Viet Nam	1.31 (1.30 to 1.33)	1.24 (1.23 to 1.25)	1.25 (1.24 to 1.26)	1.35 (1.34 to 1.36)
Zambia	1.01 (1.01 to 1.01)	0.95 (0.94 to 0.95)	1.15 (1.15 to 1.16)	1.22 (1.22 to 1.23)

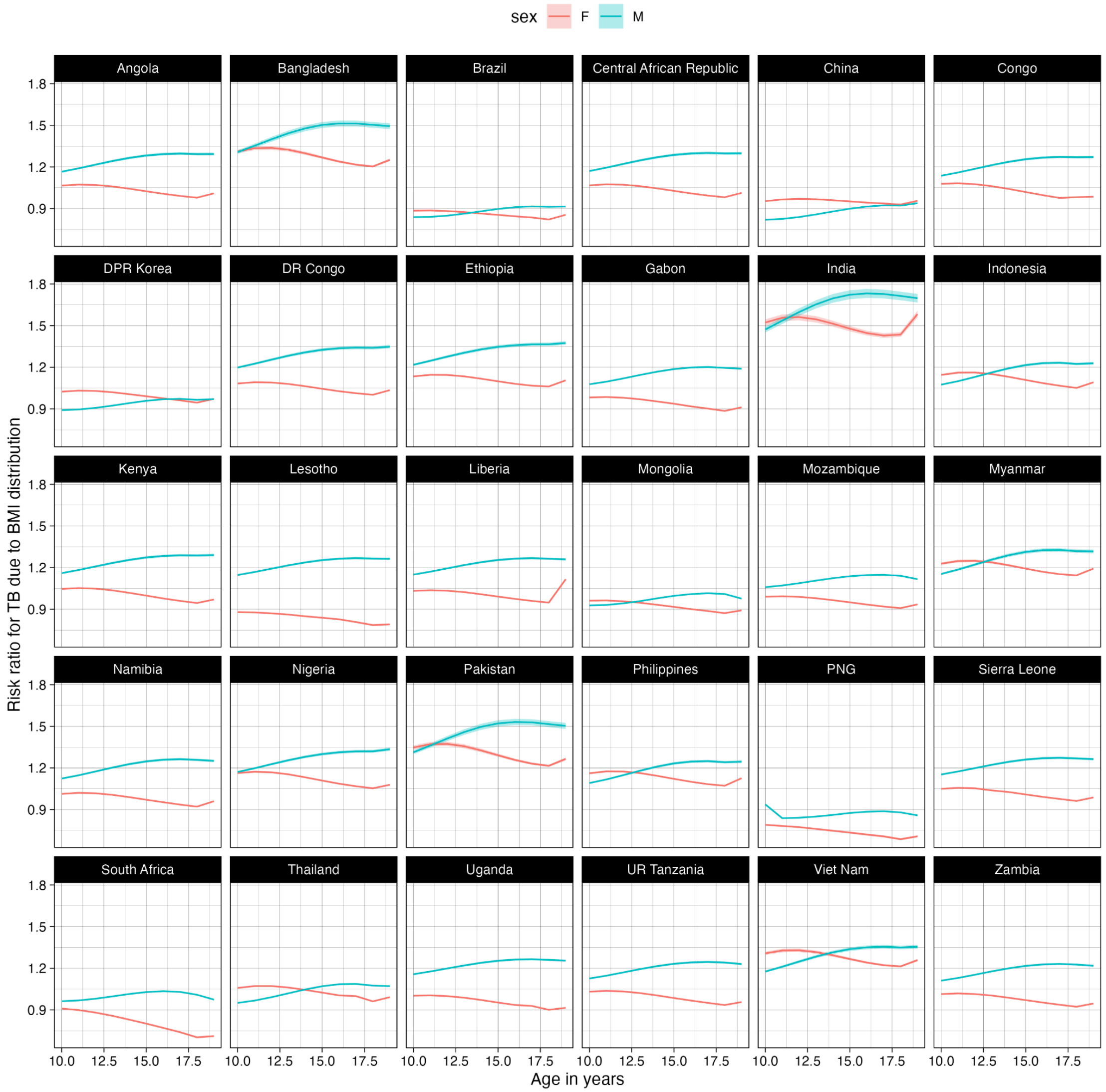

Figure S9 Estimated risk ratios for TB due to BMI by country, sex, and single year age group

Country-level TB disease incidence estimates

Table S5 TB incidence estimates by country, age, and analysis type

iso3	mixing	Risk factors	Age 10-14	Age 15-19
AGO	assortative	With risk factors	5 770 (3 170 to 10 500)	7 210 (4 010 to 13 200)
AGO	assortative	Without risk factors	5 000 (2 760 to 9 110)	5 840 (3 310 to 10 900)
AGO	random	With risk factors	5 780 (3 160 to 10 600)	5 960 (3 340 to 10 800)
AGO	random	Without risk factors	5 010 (2 750 to 9 200)	4 830 (2 760 to 8 920)
BGD	assortative	With risk factors	12 800 (6 920 to 22 400)	19 500 (10 600 to 34 000)
BGD	assortative	Without risk factors	9 430 (5 070 to 16 500)	13 800 (7 490 to 24 000)
BGD	random	With risk factors	12 800 (6 880 to 22 400)	16 900 (9 340 to 29 100)
BGD	random	Without risk factors	9 430 (5 070 to 16 500)	12 000 (6 580 to 20 600)
BRA	assortative	With risk factors	1 520 (788 to 2 720)	2 700 (1 280 to 4 390)
BRA	assortative	Without risk factors	1 760 (908 to 3 130)	3 010 (1 440 to 4 920)
BRA	random	With risk factors	1 520 (789 to 2 720)	2 100 (1 040 to 3 360)
BRA	random	Without risk factors	1 760 (909 to 3 130)	2 340 (1 160 to 3 760)
CAF	assortative	With risk factors	1 300 (676 to 2 410)	1 680 (901 to 3 060)
CAF	assortative	Without risk factors	1 100 (578 to 2 030)	1 340 (712 to 2 500)
CAF	random	With risk factors	1 300 (675 to 2 420)	1 410 (752 to 2 570)
CAF	random	Without risk factors	1 100 (578 to 2 040)	1 120 (596 to 2 080)
CHN	assortative	With risk factors	22 200 (11 800 to 38 700)	35 600 (19 000 to 60 300)
CHN	assortative	Without risk factors	24 700 (13 100 to 43 100)	38 100 (20 400 to 64 700)
CHN	random	With risk factors	22 200 (11 800 to 38 700)	28 000 (15 000 to 48 000)
CHN	random	Without risk factors	24 700 (13 200 to 43 100)	30 000 (16 000 to 51 400)

COD	assortative	With risk factors	18 500 (9 680 to 31 100)	20 500 (10 500 to 34 300)
COD	assortative	Without risk factors	15 700 (8 190 to 26 400)	16 700 (8 530 to 28 100)
COD	random	With risk factors	18 500 (9 630 to 31 100)	17 800 (9 100 to 29 300)
COD	random	Without risk factors	15 700 (8 160 to 26 300)	14 500 (7 390 to 24 100)
COG	assortative	With risk factors	936 (571 to 1 510)	1 130 (627 to 1 860)
COG	assortative	Without risk factors	803 (482 to 1 290)	925 (520 to 1 490)
COG	random	With risk factors	936 (573 to 1 510)	966 (543 to 1 570)
COG	random	Without risk factors	803 (484 to 1 290)	792 (452 to 1 270)
ETH	assortative	With risk factors	15 200 (7 940 to 26 700)	23 300 (12 000 to 45 700)
ETH	assortative	Without risk factors	12 400 (6 490 to 21 800)	18 300 (9 450 to 36 200)
ETH	random	With risk factors	15 200 (7 960 to 26 700)	17 600 (9 370 to 33 800)
ETH	random	Without risk factors	12 400 (6 510 to 21 800)	13 900 (7 380 to 26 800)
GAB	assortative	With risk factors	190 (105 to 301)	234 (111 to 452)
GAB	assortative	Without risk factors	179 (99.7 to 283)	210 (99.9 to 399)
GAB	random	With risk factors	190 (105 to 301)	204 (97.3 to 389)
GAB	random	Without risk factors	179 (99.4 to 284)	184 (87.8 to 342)
IDN	assortative	With risk factors	18 000 (10 200 to 34 600)	29 200 (15 100 to 48 700)
IDN	assortative	Without risk factors	15 700 (8 920 to 30 300)	24 800 (12 800 to 41 400)
IDN	random	With risk factors	18 000 (10 200 to 34 700)	22 800 (12 100 to 38 200)
IDN	random	Without risk factors	15 700 (8 910 to 30 400)	19 400 (10 300 to 32 500)
IND	assortative	With risk factors	195 000 (97 600 to 327 000)	315 000 (163 000 to 518 000)
IND	assortative	Without risk factors	124 000 (62 100 to 208 000)	195 000 (101 000 to 321 000)
IND	random	With risk factors	195 000 (97 900 to 326 000)	244 000 (129 000 to 394 000)
IND	random	Without risk factors	124 000 (62 300 to 208 000)	151 000 (80 400 to 245 000)
KEN	assortative	With risk factors	7 110 (3 730 to 12 000)	9 370 (4 900 to 16 200)
KEN	assortative	Without risk factors	6 120 (3 240 to 10 300)	7 780 (4 050 to 13 600)

KEN	random	With risk factors	7 110 (3 720 to 12 000)	7 600 (4 050 to 12 900)
KEN	random	Without risk factors	6 120 (3 240 to 10 300)	6 340 (3 370 to 10 900)
LBR	assortative	With risk factors	600 (298 to 1 060)	816 (473 to 1 360)
LBR	assortative	Without risk factors	534 (265 to 943)	683 (399 to 1 130)
LBR	random	With risk factors	600 (297 to 1 060)	698 (409 to 1 210)
LBR	random	Without risk factors	534 (265 to 947)	584 (346 to 1 000)
LSO	assortative	With risk factors	459 (257 to 840)	652 (368 to 1 190)
LSO	assortative	Without risk factors	409 (227 to 769)	516 (290 to 904)
LSO	random	With risk factors	459 (257 to 836)	602 (341 to 1 090)
LSO	random	Without risk factors	409 (225 to 766)	478 (268 to 853)
MMR	assortative	With risk factors	4 170 (1 990 to 7 040)	6 160 (3 100 to 10 400)
MMR	assortative	Without risk factors	3 380 (1 610 to 5 720)	4 870 (2 450 to 8 250)
MMR	random	With risk factors	4 170 (1 990 to 7 050)	5 430 (2 780 to 9 080)
MMR	random	Without risk factors	3 380 (1 610 to 5 730)	4 300 (2 210 to 7 210)
MNG	assortative	With risk factors	131 (68.2 to 227)	166 (94.2 to 263)
MNG	assortative	Without risk factors	137 (71.8 to 239)	173 (97.9 to 272)
MNG	random	With risk factors	131 (68.1 to 227)	128 (75.6 to 201)
MNG	random	Without risk factors	137 (71.7 to 239)	134 (79 to 209)
MOZ	assortative	With risk factors	5 610 (3 030 to 9 770)	7 250 (3 890 to 12 200)
MOZ	assortative	Without risk factors	4 880 (2 610 to 8 710)	5 510 (3 030 to 9 050)
MOZ	random	With risk factors	5 610 (3 040 to 9 850)	6 660 (3 580 to 11 100)
MOZ	random	Without risk factors	4 880 (2 600 to 8 760)	5 060 (2 800 to 8 250)
NAM	assortative	With risk factors	454 (250 to 725)	514 (299 to 877)
NAM	assortative	Without risk factors	391 (217 to 612)	439 (256 to 749)
NAM	random	With risk factors	454 (250 to 723)	453 (264 to 768)
NAM	random	Without risk factors	390 (216 to 608)	388 (229 to 659)

NGA	assortative	With risk factors	20 900 (11 500 to 35 700)	25 900 (13 100 to 43 500)
NGA	assortative	Without risk factors	17 400 (9 550 to 29 700)	20 700 (10 400 to 34 800)
NGA	random	With risk factors	20 900 (11 400 to 35 800)	22 300 (11 500 to 37 000)
NGA	random	Without risk factors	17 400 (9 540 to 29 800)	17 900 (9 170 to 29 700)
PAK	assortative	With risk factors	34 700 (16 200 to 62 400)	46 500 (24 600 to 79 700)
PAK	assortative	Without risk factors	25 100 (11 800 to 45 200)	32 900 (17 400 to 56 300)
PAK	random	With risk factors	34 700 (16 300 to 63 100)	39 700 (21 500 to 66 900)
PAK	random	Without risk factors	25 100 (11 800 to 45 600)	28 100 (15 200 to 47 500)
PHL	assortative	With risk factors	13 000 (7 190 to 22 400)	19 300 (10 500 to 32 000)
PHL	assortative	Without risk factors	11 200 (6 230 to 19 300)	15 800 (8 490 to 26 400)
PHL	random	With risk factors	13 000 (7 180 to 22 400)	15 400 (8 570 to 25 400)
PHL	random	Without risk factors	11 200 (6 200 to 19 300)	12 700 (6 990 to 20 800)
PNG	assortative	With risk factors	431 (241 to 771)	629 (338 to 1 100)
PNG	assortative	Without risk factors	523 (293 to 934)	765 (403 to 1 330)
PNG	random	With risk factors	431 (242 to 771)	482 (270 to 829)
PNG	random	Without risk factors	523 (293 to 936)	587 (320 to 999)
PRK	assortative	With risk factors	982 (486 to 1 730)	1 560 (857 to 2 760)
PRK	assortative	Without risk factors	1 020 (503 to 1 790)	1 610 (884 to 2 850)
PRK	random	With risk factors	982 (486 to 1 730)	1 330 (737 to 2 340)
PRK	random	Without risk factors	1 020 (503 to 1 790)	1 370 (761 to 2 410)
SLE	assortative	With risk factors	1 150 (586 to 1 940)	1 440 (770 to 2 340)
SLE	assortative	Without risk factors	1 010 (517 to 1 710)	1 210 (641 to 1 960)
SLE	random	With risk factors	1 150 (585 to 1 930)	1 260 (678 to 2 020)
SLE	random	Without risk factors	1 010 (517 to 1 710)	1 060 (567 to 1 700)
THA	assortative	With risk factors	1 840 (946 to 3 380)	2 940 (1 500 to 5 480)
THA	assortative	Without risk factors	1 790 (920 to 3 290)	2 760 (1 410 to 5 160)

THA	random	With risk factors	1 840 (947 to 3 380)	2 520 (1 320 to 4 670)
THA	random	Without risk factors	1 790 (921 to 3 290)	2 360 (1 230 to 4 380)
TZA	assortative	With risk factors	8 560 (4 650 to 14 000)	9 860 (5 140 to 17 600)
TZA	assortative	Without risk factors	7 640 (4 130 to 12 300)	8 370 (4 350 to 14 900)
TZA	random	With risk factors	8 560 (4 660 to 14 000)	8 980 (4 710 to 15 800)
TZA	random	Without risk factors	7 640 (4 130 to 12 300)	7 640 (3 990 to 13 500)
UGA	assortative	With risk factors	4 180 (2 330 to 8 450)	4 910 (2 550 to 8 100)
UGA	assortative	Without risk factors	3 660 (2 070 to 7 320)	4 160 (2 160 to 6 730)
UGA	random	With risk factors	4 180 (2 340 to 8 460)	4 450 (2 350 to 7 400)
UGA	random	Without risk factors	3 660 (2 070 to 7 330)	3 780 (1 990 to 6 170)
VNM	assortative	With risk factors	4 400 (2 320 to 7 340)	5 920 (3 310 to 10 200)
VNM	assortative	Without risk factors	3 440 (1 810 to 5 740)	4 480 (2 500 to 7 720)
VNM	random	With risk factors	4 400 (2 320 to 7 340)	5 150 (2 860 to 8 660)
VNM	random	Without risk factors	3 440 (1 810 to 5 740)	3 900 (2 180 to 6 590)
ZAF	assortative	With risk factors	8 510 (4 750 to 14 200)	10 800 (6 090 to 18 500)
ZAF	assortative	Without risk factors	8 400 (4 730 to 13 800)	10 200 (5 950 to 16 800)
ZAF	random	With risk factors	8 500 (4 750 to 14 100)	9 180 (5 120 to 15 600)
ZAF	random	Without risk factors	8 400 (4 710 to 13 700)	8 730 (5 190 to 14 300)
ZMB	assortative	With risk factors	2 880 (1 600 to 5 030)	3 660 (2 060 to 6 460)
ZMB	assortative	Without risk factors	2 510 (1 390 to 4 310)	3 000 (1 690 to 5 340)
ZMB	random	With risk factors	2 880 (1 610 to 5 010)	3 140 (1 790 to 5 490)
ZMB	random	Without risk factors	2 510 (1 400 to 4 290)	2 580 (1 480 to 4 490)
TOTAL	assortative	With risk factors	411 000 (309 000 to 550 000)	615 000 (458 000 to 823 000)
TOTAL	assortative	Without risk factors	311 000 (240 000 to 400 000)	444 000 (339 000 to 575 000)
TOTAL	random	With risk factors	411 000 (308 000 to 551 000)	493 000 (374 000 to 648 000)
TOTAL	random	Without risk factors	311 000 (240 000 to 400 000)	358 000 (280 000 to 456 000)

Country-level PAFs by sex and age

Table S6 Prevalence of risk factors and associated population attributable fractions (PAFs) for risk factors by country, sex, and age category. BMI < -2z is the prevalence of a body mass index (BMI) more than 2 standard deviations below the mean according the World Health Organization's single-year age/sex reference tables (i.e., the prevalence of being severe and moderate underweight); HIV is the HIV prevalence; ART the coverage of antiretroviral therapy among people living with HIV; PPP = pregnant or postpartum (up to 6 months). M=male; F=female.

	Adolescents 10-14 years old							Adolescents 15-19 years old							
**Country**	**BMI < -2z (%)**	**BMI PAF (%)**	**HIV (%)**	**ART (%)**	**HIV PAF (%)**	**PPP (%)**	**PPP PAF (%)**	**BMI < -2z (%)**	**BMI PAF (%)**	**HIV (%)**	**ART (%)**	**HIV PAF (%)**	**PPP (%)**	**PPP PAF (%)**	**Sex**
Angola	106.2 (105.9 to 106.5)	5.9 (5.6 to 6.1)	0.2 (0.1 to 0.2)	16.8 (16.8 to 16.8)	1 (0.4 to 1.8)	1.4 (1.4 to 1.4)	0.8 (0.5 to 1.1)	100.2 (100.1 to 100.3)	0.2 (0.1 to 0.3)	0.7 (0.4 to 1.2)	6.4 (6.4 to 6.4)	4.5 (1.8 to 9.2)	18.1 (18.1 to 18.1)	9.8 (6.4 to 13.4)	F
Angola	121.6 (120.8 to 122.4)	17.7 (17.2 to 18.3)	0.2 (0.1 to 0.2)	17 (17 to 17)	1 (0.4 to 1.9)	0 (0 to 0)	0 (0 to 0)	129.1 (128.1 to 130.1)	22.5 (21.9 to 23.2)	0.1 (0.1 to 0.2)	20.5 (20.5 to 20.5)	0.9 (0.4 to 1.8)	0 (0 to 0)	0 (0 to 0)	M
Bangladesh	132.1 (130.8 to 133.3)	24.3 (23.6 to 25)	0 (0 to 0)	77.6 (77.6 to 77.6)	0 (0 to 0)	0.3 (0.3 to 0.3)	0.2 (0.1 to 0.2)	123.6 (122.7 to 124.4)	19.1 (18.5 to 19.6)	0 (0 to 0)	30.3 (30.3 to 30.3)	0 (0 to 0)	9.9 (9.9 to 9.9)	5.6 (3.6 to 7.7)	F
Bangladesh	139.6 (137.8 to 141.4)	28.4 (27.4 to 29.3)	0 (0 to 0)	75.3 (75.3 to 75.3)	0 (0 to 0)	0 (0 to 0)	0 (0 to 0)	150.5 (148.4 to 152.6)	33.6 (32.6 to 34.4)	0 (0 to 0)	28.8 (28.8 to 28.8)	0 (0 to 0)	0 (0 to 0)	0 (0 to 0)	M
Brazil	87.8 (87.6 to 88.1)	-13.9 (-14.2 to -13.5)	0 (0 to 0)	82.5 (82.5 to 82.5)	0.1 (0 to 0.1)	0.3 (0.3 to 0.3)	0.2 (0.1 to 0.3)	84.2 (83.8 to 84.7)	-18.8 (-19.4 to -18.1)	0.1 (0.1 to 0.2)	22.3 (22.3 to 22.3)	0.6 (0.3 to 1.3)	6.4 (6.4 to 6.4)	3.6 (2.3 to 5.1)	F
Brazil	85.4 (85.1 to 85.7)	-17.1 (-17.5 to -16.6)	0 (0 to 0)	77.5 (77.5 to 77.5)	0.1 (0 to 0.1)	0 (0 to 0)	0 (0 to 0)	91 (90.8 to 91.2)	-9.9 (-10.1 to -9.7)	0.1 (0.1 to 0.1)	23.8 (23.8 to 23.8)	0.6 (0.2 to 1.1)	0 (0 to 0)	0 (0 to 0)	M
Central African Republic	106.4 (106.1 to 106.7)	6 (5.8 to 6.3)	0.5 (0.4 to 0.6)	16.1 (16.1 to 16.1)	2.9 (1.4 to 5.2)	1.3 (1.3 to 1.3)	0.8 (0.5 to 1.1)	100.5 (100.4 to 100.6)	0.5 (0.4 to 0.6)	1 (0.4 to 1.7)	28.5 (28.5 to 28.5)	5 (1.8 to 10.6)	20.6 (20.6 to 20.6)	10.9 (6.9 to 15.1)	F

Central African Republic	122.1 (121.3 to 122.9)	18.1 (17.6 to 18.6)	0.5 (0.4 to 0.6)	15.7 (15.7 to 15.7)	2.9 (1.3 to 5.2)	0 (0 to 0)	0 (0 to 0)	129.6 (128.5 to 130.6)	22.8 (22.2 to 23.4)	0.4 (0.3 to 0.5)	20.1 (20.1 to 20.1)	2.5 (1.2 to 4.4)	0 (0 to 0)	0 (0 to 0)	M
China	96.3 (96.3 to 96.4)	-3.8 (-3.9 to -3.8)	0 (0 to 0)	46 (46 to 46)	0 (0 to 0)	0 (0 to 0)	0 (0 to 0)	94.3 (94.1 to 94.5)	-6 (-6.2 to -5.8)	0 (0 to 0)	20.2 (20.2 to 20.2)	0.1 (0 to 0.1)	1 (1 to 1)	0.6 (0.4 to 0.9)	F
China	84.4 (84.1 to 84.7)	-18.5 (-18.9 to -18.1)	0 (0 to 0)	40.2 (40.2 to 40.2)	0 (0 to 0)	0 (0 to 0)	0 (0 to 0)	91.9 (91.8 to 92)	-8.8 (-8.9 to -8.7)	0 (0 to 0)	13.3 (13.3 to 13.3)	0.1 (0 to 0.3)	0 (0 to 0)	0 (0 to 0)	M
DR Congo	108.2 (107.8 to 108.5)	7.6 (7.2 to 7.9)	0.1 (0.1 to 0.2)	28.7 (28.7 to 28.7)	0.7 (0.3 to 1.3)	0.6 (0.6 to 0.6)	0.3 (0.2 to 0.5)	102.4 (102.3 to 102.5)	2.4 (2.3 to 2.5)	0.2 (0.1 to 0.3)	74.7 (74.7 to 74.7)	0.5 (0.2 to 1.1)	13.9 (13.9 to 13.9)	7.7 (5 to 10.3)	F
DR Congo	125.4 (124.5 to 126.3)	20.2 (19.7 to 20.8)	0.1 (0.1 to 0.2)	28 (28 to 28)	0.7 (0.3 to 1.2)	0 (0 to 0)	0 (0 to 0)	133.9 (132.7 to 135.1)	25.3 (24.6 to 26)	0.1 (0.1 to 0.2)	100 (100 to 100)	0.2 (0 to 0.4)	0 (0 to 0)	0 (0 to 0)	M
Congo	106.8 (106.5 to 107)	6.3 (6.1 to 6.6)	0.5 (0.4 to 0.6)	14.8 (14.8 to 14.8)	3.1 (1.4 to 5.5)	0.6 (0.6 to 0.6)	0.4 (0.2 to 0.5)	99.2 (99.1 to 99.3)	-0.8 (-0.9 to -0.7)	0.9 (0.4 to 1.5)	9.9 (9.9 to 9.9)	5.7 (2.2 to 11.3)	14.3 (14.3 to 14.3)	7.9 (5.3 to 10.8)	F
Congo	118.7 (118 to 119.4)	15.7 (15.2 to 16.2)	0.5 (0.4 to 0.7)	14.1 (14.1 to 14.1)	3.2 (1.5 to 5.8)	0 (0 to 0)	0 (0 to 0)	126.7 (125.8 to 127.7)	21.1 (20.5 to 21.7)	0.4 (0.3 to 0.5)	21.3 (21.3 to 21.3)	2.4 (1.2 to 4.4)	0 (0 to 0)	0 (0 to 0)	M
Ethiopia	113.5 (113 to 114)	11.9 (11.5 to 12.3)	0.3 (0.2 to 0.4)	29.9 (29.9 to 29.9)	1.6 (0.7 to 2.9)	0.1 (0.1 to 0.1)	0.1 (0 to 0.1)	108.3 (108 to 108.6)	7.7 (7.4 to 7.9)	0.4 (0.3 to 0.4)	42.1 (42.1 to 42.1)	1.6 (0.7 to 3)	9.3 (9.3 to 9.3)	5.2 (3.3 to 7.2)	F
Ethiopia	127.5 (126.5 to 128.5)	21.6 (21 to 22.1)	0.3 (0.2 to 0.4)	30.4 (30.4 to 30.4)	1.6 (0.7 to 2.8)	0 (0 to 0)	0 (0 to 0)	136.3 (135 to 137.6)	26.6 (25.9 to 27.3)	0.3 (0.2 to 0.3)	53 (53 to 53)	1.1 (0.5 to 2.1)	0 (0 to 0)	0 (0 to 0)	M
Gabon	97.4 (97.4 to 97.5)	-2.6 (-2.7 to -2.6)	0.3 (0.2 to 0.4)	67.8 (67.8 to 67.8)	1.1 (0.4 to 2.1)	0.4 (0.4 to 0.4)	0.3 (0.2 to 0.4)	91.1 (90.8 to 91.4)	-9.8 (-10.1 to -9.5)	0.8 (0.4 to 1.4)	31.7 (31.7 to 31.7)	4.3 (1.6 to 8.7)	12.2 (12.2 to 12.2)	6.8 (4.5 to 9.2)	F
Gabon	112.1 (111.6 to 112.6)	10.8 (10.4 to 11.2)	0.3 (0.3 to 0.4)	64.8 (64.8 to 64.8)	1.2 (0.5 to 2.2)	0 (0 to 0)	0 (0 to 0)	119.4 (118.7 to 120.1)	16.3 (15.8 to 16.7)	0.3 (0.2 to 0.4)	95.1 (95.1 to 95.1)	0.5 (0.1 to 1.1)	0 (0 to 0)	0 (0 to 0)	M
Indonesia	115 (114.3 to 115.7)	13 (12.5 to 13.5)	0 (0 to 0)	18.5 (18.5 to 18.5)	0.1 (0 to 0.1)	0 (0 to 0)	0 (0 to 0)	108.1 (107.8 to 108.4)	7.5 (7.2 to 7.8)	0.1 (0.1 to 0.1)	2.4 (2.4 to 2.4)	0.6 (0.3 to 1.2)	3.6 (3.6 to 3.6)	2.1 (1.4 to 2.9)	F
Indonesia	113.2 (112.6 to 113.8)	11.7 (11.2 to 12.2)	0 (0 to 0)	43.5 (43.5 to 43.5)	0 (0 to 0.1)	0 (0 to 0)	0 (0 to 0)	122.6 (121.6 to 123.5)	18.4 (17.8 to 19)	0.1 (0.1 to 0.1)	2.3 (2.3 to 2.3)	0.6 (0.3 to 1.1)	0 (0 to 0)	0 (0 to 0)	M

India	154 (151.7 to 156.2)	35 (34.1 to 36)	0 (0 to 0)	80.8 (80.8 to 80.8)	0 (0 to 0.1)	0 (0 to 0)	0 (0 to 0)	147.4 (145.5 to 149.2)	32.1 (31.3 to 33)	0 (0 to 0)	64.6 (64.6 to 64.6)	0.1 (0 to 0.2)	1.8 (1.8 to 1.8)	1.1 (0.7 to 1.5)	F
India	159.1 (156.4 to 162)	37.2 (36.1 to 38.3)	0 (0 to 0)	78.3 (78.3 to 78.3)	0 (0 to 0.1)	0 (0 to 0)	0 (0 to 0)	171.8 (168.5 to 175.4)	41.8 (40.6 to 43)	0 (0 to 0)	86 (86 to 86)	0.1 (0 to 0.1)	0 (0 to 0)	0 (0 to 0)	M
Kenya	104 (103.8 to 104.2)	3.9 (3.6 to 4.1)	0.8 (0.7 to 1)	57.8 (57.8 to 57.8)	3.1 (1.4 to 5.5)	0.1 (0.1 to 0.1)	0.1 (0.1 to 0.1)	97 (96.9 to 97.1)	-3.1 (-3.2 to -3)	1.4 (1.2 to 1.7)	59.9 (59.9 to 59.9)	5.1 (2.2 to 9.2)	8 (8 to 8)	4.6 (2.9 to 6.4)	F
Kenya	120.8 (120 to 121.6)	17.2 (16.7 to 17.7)	0.9 (0.7 to 1)	55.6 (55.6 to 55.6)	3.3 (1.4 to 6)	0 (0 to 0)	0 (0 to 0)	128.5 (127.5 to 129.4)	22.2 (21.6 to 22.7)	0.9 (0.8 to 1)	63 (63 to 63)	3.2 (1.3 to 5.8)	0 (0 to 0)	0 (0 to 0)	M
Liberia	102.7 (102.5 to 102.8)	2.6 (2.5 to 2.7)	0.1 (0.1 to 0.2)	22.8 (22.8 to 22.8)	0.8 (0.4 to 1.5)	0.6 (0.6 to 0.6)	0.3 (0.2 to 0.5)	99.8 (99.6 to 99.9)	-0.2 (-0.4 to -0.1)	0.3 (0.2 to 0.5)	28.8 (28.8 to 28.8)	1.8 (0.7 to 3.4)	16.5 (16.5 to 16.5)	9.1 (5.9 to 12.3)	F
Liberia	119.5 (118.7 to 120.1)	16.3 (15.8 to 16.8)	0.1 (0.1 to 0.2)	21.8 (21.8 to 21.8)	0.8 (0.4 to 1.5)	0 (0 to 0)	0 (0 to 0)	126.2 (125.4 to 127.1)	20.8 (20.2 to 21.3)	0.1 (0.1 to 0.2)	31.8 (31.8 to 31.8)	0.6 (0.3 to 1.1)	0 (0 to 0)	0 (0 to 0)	M
Lesotho	86.8 (86.4 to 87.1)	-15.3 (-15.7 to -14.8)	3 (2.7 to 3.4)	73.7 (73.7 to 73.7)	8.2 (3.4 to 14.7)	0.1 (0.1 to 0.1)	0.1 (0 to 0.1)	81 (80.5 to 81.5)	-23.4 (-24.3 to -22.6)	6.2 (4.4 to 8.2)	39.9 (39.9 to 39.9)	22.8 (11.9 to 37.3)	9.6 (9.6 to 9.6)	5.4 (3.4 to 7.6)	F
Lesotho	119.2 (118.6 to 119.9)	16.1 (15.7 to 16.6)	3.2 (2.7 to 3.6)	74.2 (74.2 to 74.2)	8.4 (3.5 to 15.2)	0 (0 to 0)	0 (0 to 0)	126.3 (125.4 to 127.1)	20.8 (20.3 to 21.3)	2.9 (2.1 to 3.7)	60.3 (60.3 to 60.3)	9.7 (4.2 to 17.2)	0 (0 to 0)	0 (0 to 0)	M
Myanmar	123.6 (122.7 to 124.5)	19.1 (18.5 to 19.7)	0.1 (0.1 to 0.2)	53.9 (53.9 to 53.9)	0.4 (0.2 to 0.9)	0.1 (0.1 to 0.1)	0 (0 to 0.1)	117.1 (116.4 to 117.7)	14.6 (14.1 to 15)	0.1 (0.1 to 0.2)	98.5 (98.5 to 98.5)	0.2 (0 to 0.4)	4.3 (4.3 to 4.3)	2.5 (1.6 to 3.5)	F
Myanmar	122.2 (121.1 to 123.2)	18.2 (17.5 to 18.9)	0.1 (0.1 to 0.2)	51.7 (51.7 to 51.7)	0.5 (0.2 to 0.9)	0 (0 to 0)	0 (0 to 0)	132 (130.7 to 133.4)	24.2 (23.5 to 25)	0.1 (0.1 to 0.1)	75.1 (75.1 to 75.1)	0.2 (0.1 to 0.5)	0 (0 to 0)	0 (0 to 0)	M
Mongolia	95.2 (95.1 to 95.3)	-5 (-5.2 to -4.9)	0 (0 to 0)	100 (100 to 100)	0 (0 to 0)	0 (0 to 0)	0 (0 to 0)	89.4 (89 to 89.7)	-11.9 (-12.4 to -11.5)	0 (0 to 0)	100 (100 to 100)	0 (0 to 0)	3.7 (3.7 to 3.7)	2.2 (1.4 to 3.1)	F
Mongolia	94.7 (94.6 to 94.9)	-5.5 (-5.7 to -5.4)	0 (0 to 0)	100 (100 to 100)	0 (0 to 0)	0 (0 to 0)	0 (0 to 0)	100.1 (100.1 to 100.2)	0.1 (0.1 to 0.2)	0 (0 to 0)	100 (100 to 100)	0 (0 to 0)	0 (0 to 0)	0 (0 to 0)	M
Mozambique	98.3 (98.3 to 98.4)	-1.7 (-1.8 to -1.6)	1.9 (1.6 to 2.3)	26.6 (26.6 to 26.6)	9.6 (4.8 to 16.5)	1 (1 to 1)	0.6 (0.4 to 0.9)	92.9 (92.7 to 93.2)	-7.6 (-7.9 to -7.4)	5.6 (3.4 to 8.4)	23.7 (23.7 to 23.7)	23.8 (11.6 to 39.6)	20.2 (20.2 to 20.2)	10.7 (7 to 14.6)	F

Mozambique	109 (108.7 to 109.2)	8.2 (8 to 8.5)	1.9 (1.6 to 2.3)	26.4 (26.4 to 26.4)	9.6 (4.7 to 16.5)	0 (0 to 0)	0 (0 to 0)	113.8 (113.4 to 114.2)	12.1 (11.8 to 12.4)	2 (1.1 to 3.2)	29.5 (29.5 to 29.5)	9.7 (4.1 to 18.7)	0 (0 to 0)	0 (0 to 0)	M
Namibia	100.9 (100.7 to 101.1)	0.9 (0.7 to 1.1)	2 (1.8 to 2.3)	71.5 (71.5 to 71.5)	5.9 (2.5 to 10.7)	0.3 (0.3 to 0.3)	0.2 (0.1 to 0.3)	94.8 (94.7 to 94.9)	-5.5 (-5.6 to -5.3)	2.5 (2 to 3)	91.2 (91.2 to 91.2)	4.7 (1.5 to 9.7)	9 (9 to 9)	5.1 (3.3 to 7.1)	F
Namibia	117.5 (116.8 to 118.2)	14.9 (14.4 to 15.4)	2.1 (1.8 to 2.5)	69 (69 to 69)	6.3 (2.6 to 11.2)	0 (0 to 0)	0 (0 to 0)	125.6 (124.7 to 126.4)	20.4 (19.8 to 20.9)	1.6 (1.3 to 1.9)	95.4 (95.4 to 95.4)	2.6 (0.7 to 5.8)	0 (0 to 0)	0 (0 to 0)	M
Nigeria	115.8 (115.2 to 116.4)	13.6 (13.2 to 14.1)	0.1 (0.1 to 0.1)	59.2 (59.2 to 59.2)	0.4 (0.2 to 0.7)	0.4 (0.4 to 0.4)	0.3 (0.2 to 0.4)	107.9 (107.6 to 108.2)	7.3 (7.1 to 7.6)	0.3 (0.2 to 0.4)	54.4 (54.4 to 54.4)	1.1 (0.5 to 2.1)	11.6 (11.6 to 11.6)	6.5 (4.1 to 9.1)	F
Nigeria	122.6 (121.7 to 123.5)	18.4 (17.9 to 19)	0.1 (0.1 to 0.1)	58.3 (58.3 to 58.3)	0.4 (0.2 to 0.8)	0 (0 to 0)	0 (0 to 0)	131.7 (130.6 to 132.9)	24.1 (23.5 to 24.7)	0.1 (0.1 to 0.1)	75.7 (75.7 to 75.7)	0.3 (0.1 to 0.6)	0 (0 to 0)	0 (0 to 0)	M
Pakistan	135.4 (134 to 137)	26.2 (25.4 to 27)	0 (0 to 0)	28.8 (28.8 to 28.8)	0 (0 to 0)	0.1 (0.1 to 0.1)	0 (0 to 0)	125.2 (124.2 to 126.1)	20.1 (19.5 to 20.7)	0 (0 to 0.1)	3.4 (3.4 to 3.4)	0.1 (0 to 0.4)	5.7 (5.7 to 5.7)	3.3 (2.1 to 4.6)	F
Pakistan	140.8 (138.9 to 142.7)	29 (28 to 29.9)	0 (0 to 0)	26.3 (26.3 to 26.3)	0 (0 to 0)	0 (0 to 0)	0 (0 to 0)	152 (149.8 to 154.4)	34.2 (33.3 to 35.2)	0 (0 to 0.1)	2 (2 to 2)	0.1 (0 to 0.7)	0 (0 to 0)	0 (0 to 0)	M
Philippines	116.3 (115.7 to 117)	14 (13.6 to 14.5)	0 (0 to 0)	8 (8 to 8)	0.1 (0 to 0.1)	0.1 (0.1 to 0.1)	0 (0 to 0)	110.1 (109.7 to 110.4)	9.1 (8.9 to 9.4)	0.2 (0.1 to 0.3)	0.5 (0.5 to 0.5)	1.3 (0.4 to 2.8)	4.8 (4.8 to 4.8)	2.8 (1.8 to 3.9)	F
Philippines	114.9 (114.2 to 115.6)	13 (12.4 to 13.5)	0 (0 to 0)	7.4 (7.4 to 7.4)	0.1 (0 to 0.2)	0 (0 to 0)	0 (0 to 0)	124.3 (123.3 to 125.3)	19.5 (18.9 to 20.2)	0.4 (0.2 to 0.7)	4.1 (4.1 to 4.1)	2.6 (1 to 5.7)	0 (0 to 0)	0 (0 to 0)	M
PNG	77.1 (76.5 to 77.6)	-29.8 (-30.7 to -28.8)	0.1 (0 to 0.3)	39.9 (39.9 to 39.9)	0.7 (0.1 to 2)	0.2 (0.2 to 0.2)	0.1 (0.1 to 0.2)	71.1 (70.3 to 71.9)	-40.7 (-42.3 to -39.1)	0.3 (0 to 1)	17 (17 to 17)	2 (0.1 to 7)	7.1 (7.1 to 7.1)	4.1 (2.6 to 5.7)	F
PNG	86.5 (86.2 to 86.9)	-15.6 (-16 to -15.1)	0.1 (0 to 0.3)	36.7 (36.7 to 36.7)	0.6 (0.1 to 1.8)	0 (0 to 0)	0 (0 to 0)	87.7 (87.4 to 88)	-14 (-14.4 to -13.6)	0.1 (0 to 0.3)	51 (51 to 51)	0.4 (0.1 to 1.3)	0 (0 to 0)	0 (0 to 0)	M
DPR Korea	102.2 (102 to 102.4)	2.2 (2 to 2.4)	0 (0 to 0)	97.1 (97.1 to 97.1)	0 (0 to 0.1)	0 (0 to 0)	0 (0 to 0)	96.9 (96.8 to 97)	-3.2 (-3.3 to -3.1)	0 (0 to 0.1)	33.1 (33.1 to 33.1)	0.1 (0 to 0.4)	0.1 (0.1 to 0.1)	0 (0 to 0.1)	F
DPR Korea	91.2 (91.1 to 91.3)	-9.6 (-9.8 to -9.5)	0 (0 to 0)	86.1 (86.1 to 86.1)	0 (0 to 0.1)	0 (0 to 0)	0 (0 to 0)	96.7 (96.7 to 96.8)	-3.4 (-3.4 to -3.3)	0 (0 to 0.1)	18.9 (18.9 to 18.9)	0.2 (0 to 0.9)	0 (0 to 0)	0 (0 to 0)	M

Sierra Leone	104.5 (104.2 to 104.7)	4.3 (4.1 to 4.5)	0.2 (0.1 to 0.2)	13.5 (13.5 to 13.5)	1 (0.4 to 1.7)	0.5 (0.5 to 0.5)	0.3 (0.2 to 0.4)	98.5 (98.5 to 98.6)	-1.5 (-1.6 to -1.4)	0.6 (0.3 to 0.9)	37.8 (37.8 to 37.8)	2.9 (1.2 to 5.8)	12.8 (12.8 to 12.8)	7.1 (4.6 to 9.8)	F
Sierra Leone	119.8 (119.1 to 120.6)	16.6 (16 to 17)	0.2 (0.1 to 0.2)	13.3 (13.3 to 13.3)	1 (0.5 to 1.7)	0 (0 to 0)	0 (0 to 0)	126.7 (125.8 to 127.6)	21.1 (20.5 to 21.7)	0.2 (0.1 to 0.3)	31.3 (31.3 to 31.3)	1 (0.3 to 2)	0 (0 to 0)	0 (0 to 0)	M
Thailand	106.2 (105.7 to 106.6)	5.8 (5.4 to 6.2)	0 (0 to 0.1)	100 (100 to 100)	0 (0 to 0.1)	0.1 (0.1 to 0.1)	0.1 (0 to 0.1)	99.6 (99.5 to 99.7)	-0.4 (-0.5 to -0.3)	0.2 (0.2 to 0.3)	27.8 (27.8 to 27.8)	1.3 (0.6 to 2.6)	3.9 (3.9 to 3.9)	2.3 (1.4 to 3.2)	F
Thailand	99.5 (99.2 to 99.8)	-0.5 (-0.8 to -0.2)	0 (0 to 0)	100 (100 to 100)	0 (0 to 0.1)	0 (0 to 0)	0 (0 to 0)	107.8 (107.3 to 108.3)	7.2 (6.8 to 7.6)	0.3 (0.2 to 0.4)	29.3 (29.3 to 29.3)	1.4 (0.6 to 2.6)	0 (0 to 0)	0 (0 to 0)	M
UR Tanzania	102.5 (102.4 to 102.7)	2.5 (2.3 to 2.6)	0.5 (0.5 to 0.6)	64.1 (64.1 to 64.1)	1.9 (0.8 to 3.5)	0.2 (0.2 to 0.2)	0.1 (0.1 to 0.2)	95.9 (95.8 to 96)	-4.3 (-4.4 to -4.1)	1 (0.8 to 1.3)	69.4 (69.4 to 69.4)	3.2 (1.2 to 6.2)	14.8 (14.8 to 14.8)	8.1 (5.4 to 11.2)	F
UR Tanzania	117 (116.4 to 117.7)	14.6 (14.1 to 15)	0.6 (0.5 to 0.6)	61.8 (61.8 to 61.8)	2 (0.8 to 3.6)	0 (0 to 0)	0 (0 to 0)	123.8 (123 to 124.6)	19.2 (18.7 to 19.8)	0.7 (0.5 to 0.9)	61.5 (61.5 to 61.5)	2.4 (1 to 4.8)	0 (0 to 0)	0 (0 to 0)	M
Uganda	99.3 (99.2 to 99.4)	-0.7 (-0.8 to -0.6)	0.9 (0.8 to 1)	48.5 (48.5 to 48.5)	3.8 (1.7 to 6.8)	0.2 (0.2 to 0.2)	0.1 (0.1 to 0.2)	92.6 (92.4 to 92.9)	-7.9 (-8.2 to -7.6)	1.6 (1.1 to 2)	60.4 (60.4 to 60.4)	5.5 (2.2 to 10)	14.4 (14.4 to 14.4)	8 (5.1 to 10.9)	F
Uganda	119.9 (119.2 to 120.6)	16.6 (16.1 to 17.1)	0.9 (0.8 to 1.1)	47.4 (47.4 to 47.4)	3.9 (1.7 to 6.9)	0 (0 to 0)	0 (0 to 0)	125.9 (125.1 to 126.8)	20.6 (20.1 to 21.1)	0.8 (0.6 to 0.9)	70 (70 to 70)	2.4 (0.9 to 4.6)	0 (0 to 0)	0 (0 to 0)	M
Viet Nam	131.5 (130.3 to 132.8)	23.9 (23.3 to 24.7)	0 (0 to 0)	100 (100 to 100)	0 (0 to 0)	0 (0 to 0)	0 (0 to 0)	124 (123.3 to 124.8)	19.4 (18.9 to 19.9)	0 (0 to 0)	32.6 (32.6 to 32.6)	0.1 (0.1 to 0.2)	4.4 (4.4 to 4.4)	2.6 (1.7 to 3.6)	F
Viet Nam	124.7 (123.6 to 125.7)	19.8 (19.1 to 20.5)	0 (0 to 0)	100 (100 to 100)	0 (0 to 0)	0 (0 to 0)	0 (0 to 0)	135 (133.6 to 136.4)	25.9 (25.2 to 26.7)	0.1 (0.1 to 0.2)	5.5 (5.5 to 5.5)	0.9 (0.4 to 1.9)	0 (0 to 0)	0 (0 to 0)	M
South Africa	87.5 (87.3 to 87.8)	-14.2 (-14.5 to -14)	2.2 (1.9 to 2.5)	56.8 (56.8 to 56.8)	7.8 (3.7 to 13.4)	0.1 (0.1 to 0.1)	0.1 (0 to 0.1)	74.5 (73.9 to 75.1)	-34.3 (-35.2 to -33.2)	5.6 (4.6 to 6.8)	35.3 (35.3 to 35.3)	22 (11.9 to 34.6)	6.7 (6.7 to 6.7)	3.8 (2.3 to 5.3)	F
South Africa	98.5 (98.4 to 98.6)	-1.6 (-1.7 to -1.4)	2.2 (2 to 2.5)	53.9 (53.9 to 53.9)	8.3 (3.8 to 14.3)	0 (0 to 0)	0 (0 to 0)	101.5 (101.4 to 101.7)	1.5 (1.3 to 1.7)	2.1 (1.9 to 2.4)	45.9 (45.9 to 45.9)	8.7 (4.2 to 15.2)	0 (0 to 0)	0 (0 to 0)	M

Zambia	100.8 (100.7 to 100.9)	0.8 (0.7 to 0.9)	1.4 (1.2 to 1.6)	46.5 (46.5 to 46.5)	5.8 (2.7 to 10.3)	0.4 (0.4 to 0.4)	0.2 (0.1 to 0.3)	94.6 (94.4 to 94.8)	-5.7 (-5.9 to -5.5)	2.9 (2.2 to 3.8)	59.8 (59.8 to 59.8)	9.8 (4.5 to 18)	15.4 (15.4 to 15.4)	8.4 (5.5 to 11.4)	F
Zambia	115.5 (114.9 to 116)	13.4 (13 to 13.8)	1.4 (1.2 to 1.6)	46.3 (46.3 to 46.3)	5.9 (2.7 to 10.3)	0 (0 to 0)	0 (0 to 0)	122.4 (121.6 to 123.2)	18.3 (17.8 to 18.8)	1.6 (1.1 to 2.1)	61.2 (61.2 to 61.2)	5.5 (2.2 to 10)	0 (0 to 0)	0 (0 to 0)	M

Comparison between methods

Figure S10 shows of our method variants affect comparisons with notifications, stratified by sex. Figure S11 shows directly the relative effect of method variations on country TB incidence estimates.

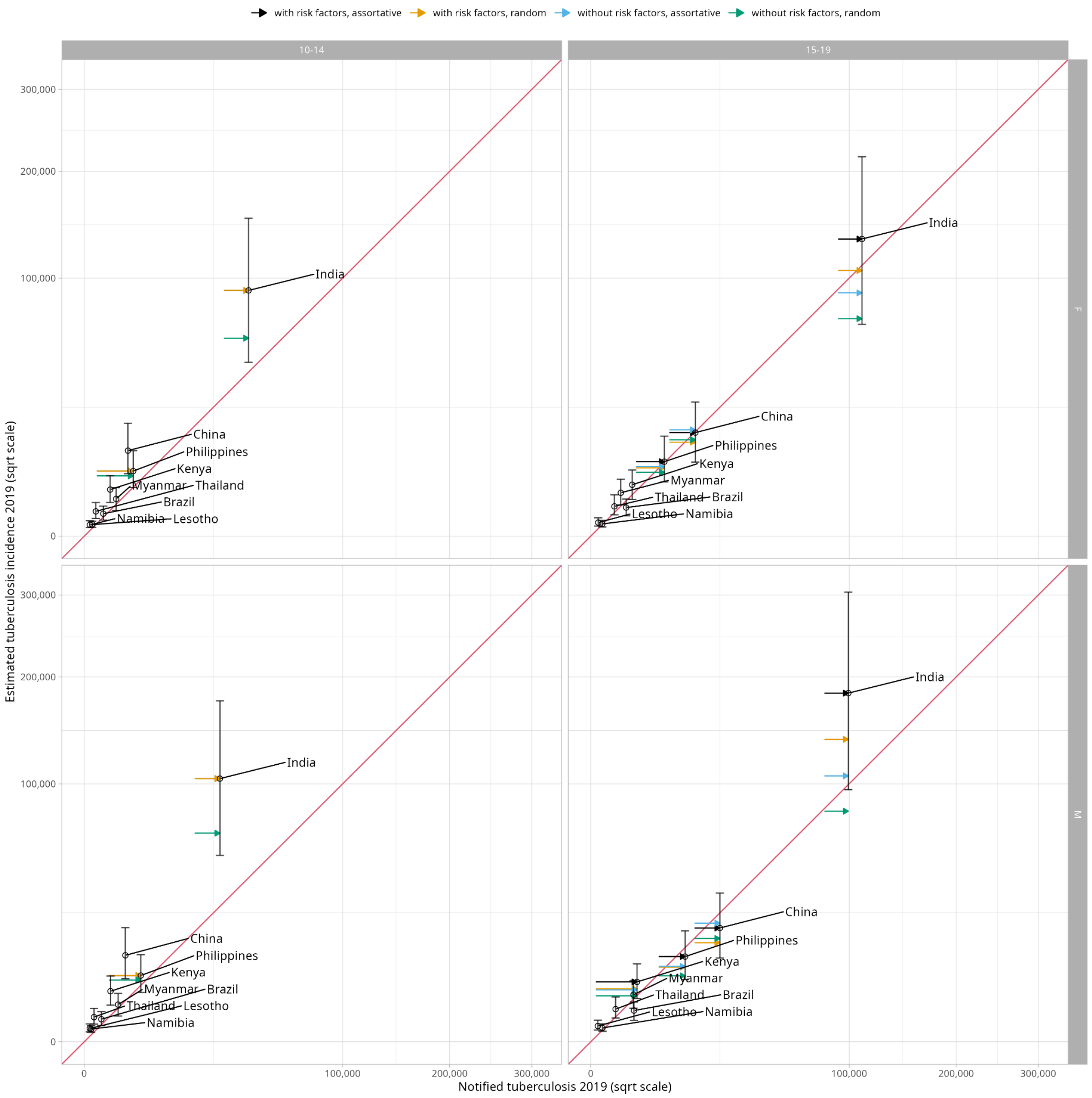

Figure S10 An alternative visualization comparing our 4 methods with notifications in each category. Red line denotes equality

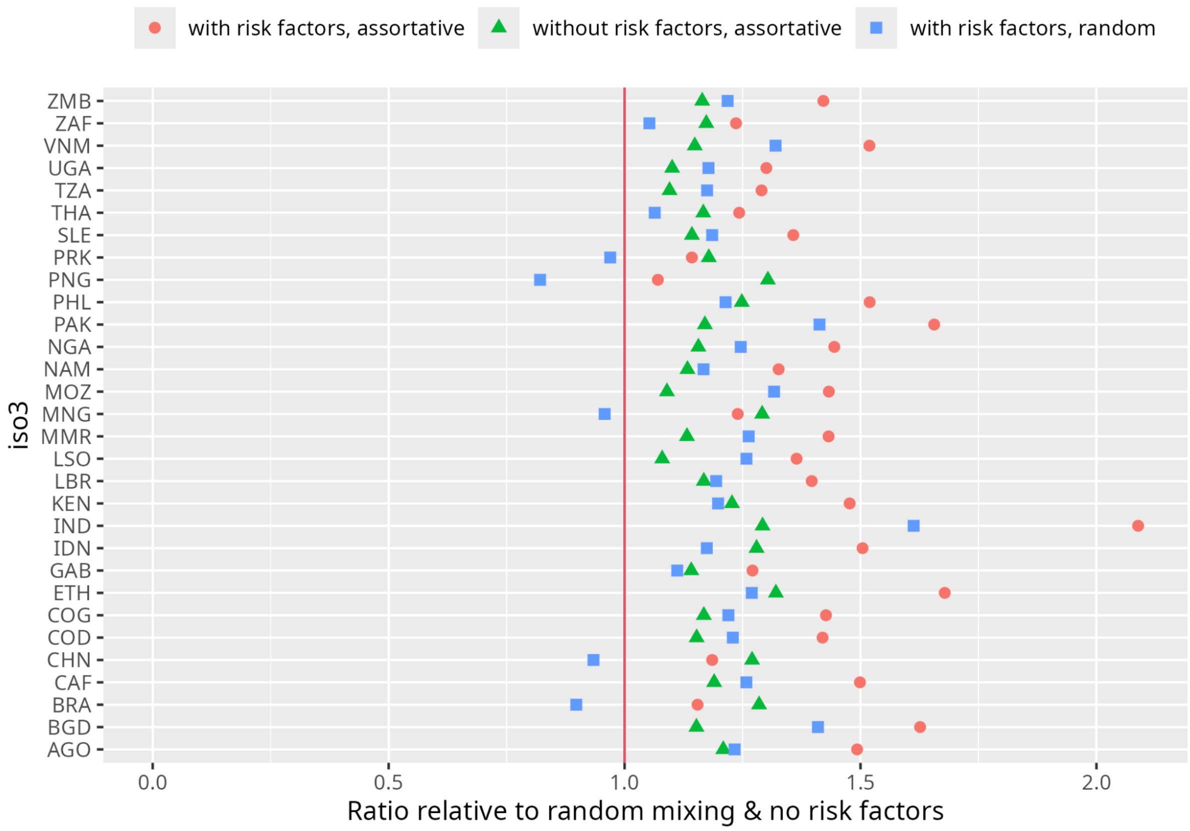

Figure S11 The ratio of incidence estimates in 15-19 year olds with the estimate assuming random mixing and no risk factors

Contribution of progression vs infection differences to age differences in TB incidence

Figure S12 compares TB disease incidence and TB infection incidence between older and younger adolescents by country, showing the degree to which risk factor differences between age groups contribute on top of differences in infection risk to generate incidence differences.

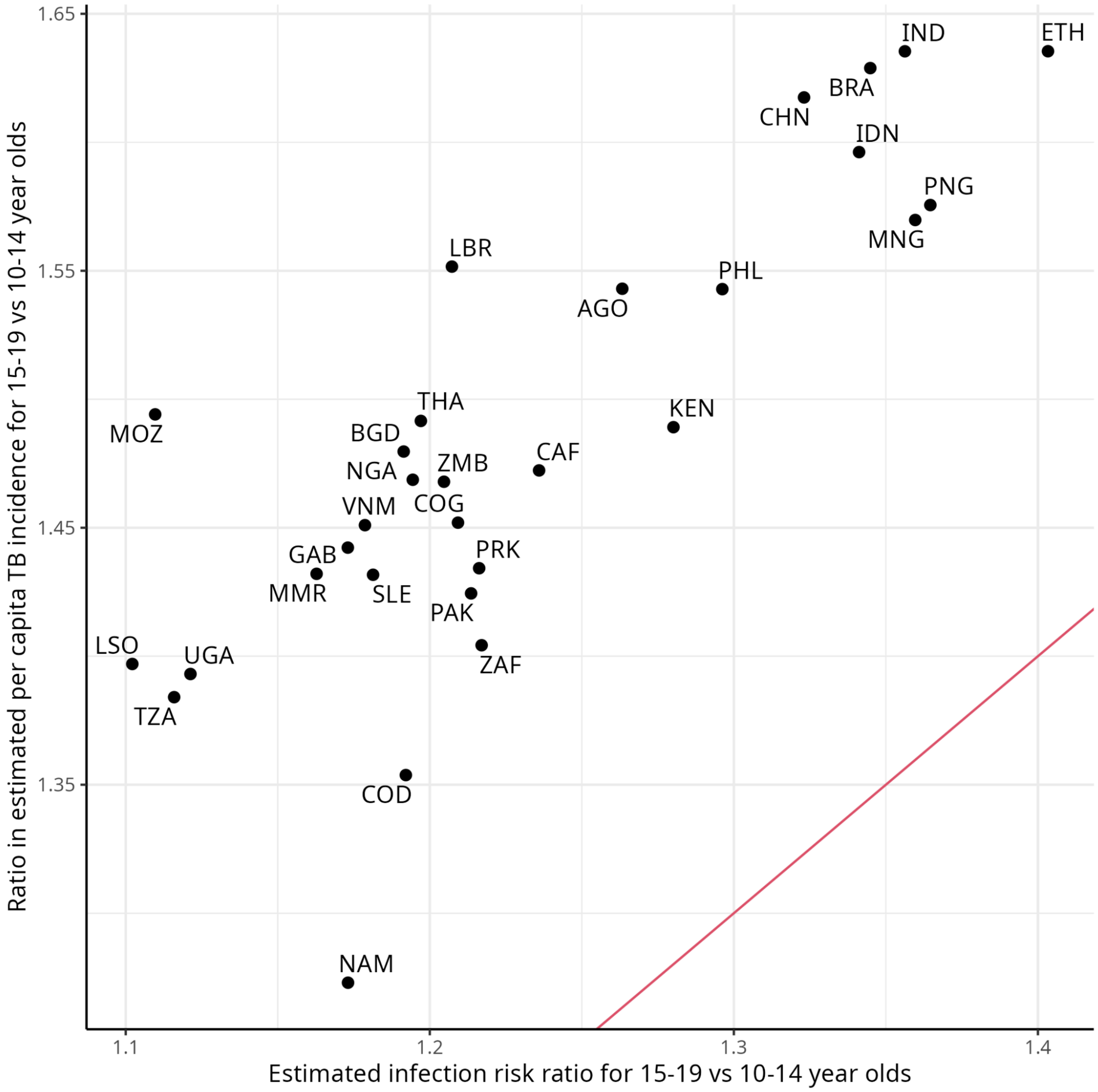

Figure S12 The ratio of estimated per capita incidence in 15-19 year olds to 10-14 year olds compared with the estimated infection risk ratio between these groups. Red line represents y=x

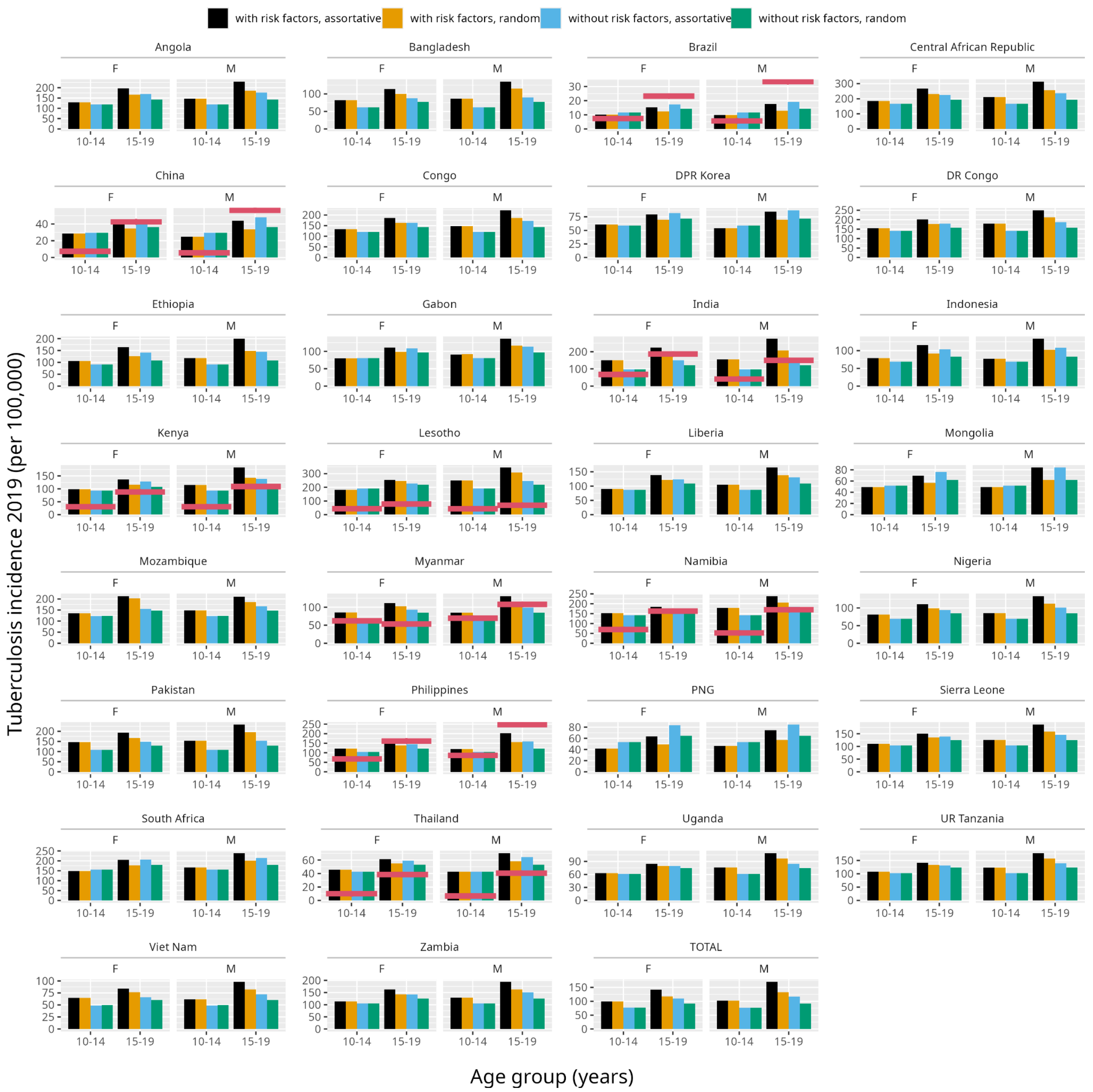

Figure S13 Per capita TB incidence by method and sex, compared with notifications (red bar). This is a per capita version of Figure 2 in the main article.

References

1 Prem K, van Zandvoort K, Klepac P, *et al.* Projecting contact matrices in 177 geographical regions: An update and comparison with empirical data for the COVID-19 era. *PLoS Comput Biol* 2021; **17**: e1009098.

2 Houben RMGJ, Dodd PJ. The Global Burden of Latent Tuberculosis Infection: A Re-estimation Using Mathematical Modelling. *PLoS Med* 2016; **13**: e1002152.

3 Lönnroth K, Williams BG, Cegielski P, Dye C. A consistent log-linear relationship between tuberculosis incidence and body mass index. *Int J Epidemiol* 2010; **39**: 149–55.

4 Dodd PJ, Prendergast AJ, Beecroft C, Kampmann B, Seddon JA. The impact of HIV and antiretroviral therapy on TB risk in children: a systematic review and meta-analysis. *Thorax* 2017; **72**: 559–75.

5 Zenner D, Kruijshaar ME, Andrews N, Abubakar I. Risk of tuberculosis in pregnancy: a national, primary care-based cohort and self-controlled case series study. *Am J Respir Crit Care Med* 2012; **185**: 779–84.

6 Jonsson J, Kühlmann-Berenzon S, Berggren I, Bruchfeld J. Increased risk of active tuberculosis during pregnancy and postpartum: a register-based cohort study in Sweden. *Eur Respir J* 2020; **55**. DOI:10.1183/13993003.01886-2019.

7 Martinez L, Cords O, Robert Horsburgh C, *et al.* The risk of tuberculosis in children after close exposure: a systematic review and individual-participant meta-analysis. *Lancet* 2020; **395**: 973–84.

8 Ragonnet R, Trauer JM, Scott N, Meehan MT, Denholm JT, McBryde ES. Optimally capturing latency dynamics in models of tuberculosis transmission. *Epidemics* 2017; **21**: 39–47.
