# Supplementary material for: Factors driving adolescent tuberculosis incidence by age and sex in 30 high-tuberculosis burden countries: a mathematical modelling study
Source: BMJ Glob Health. 2025 Mar 5;10(3):e015368. doi: 10.1136/bmjgh-2024-015368 (PMC11883532; doi:10.1136/bmjgh-2024-015368)
Supplement: online supplemental table 1 [file bmjgh-10-3-s002.pdf]

Supplementary Table 1 Prevalence of risk factors and associated population attributable fractions (PAFs). BMI < -2z is the prevalence of a body mass index (BMI) more than 2 standard deviations below the mean according the World Health Organization's single-year age/sex reference tables (i.e., the prevalence of being severe and moderate underweight); HIV is the HIV prevalence; ART the coverage of antiretroviral therapy among people living with HIV; PPP = pregnant or postpartum period (up to 6 months after delivery).

	Adolescents 10-14 years old							Adolescents 15-19 years old						
**Country**	**BMI < -2z (%)**	**BMI PAF (%)**	**HIV (%)**	**ART (%)**	**HIV PAF (%)**	**PPP (%)**	**PPP PAF (%)**	**BMI < -2z (%)**	**BMI PAF (%)**	**HIV (%)**	**ART (%)**	**HIV PAF (%)**	**PPP (%)**	**PPP PAF (%)**
Angola	9.7 (0 to 20.7)	11.8 (5.6 to 18.2)	0.2 (0.1 to 0.2)	16.9 (16.8 to 17)	1 (0.4 to 1.9)	0.7 (0 to 1.4)	0.4 (0 to 1.1)	6.7 (0 to 16.1)	11.4 (0.1 to 23.1)	0.4 (0.1 to 1.1)	13.4 (6.4 to 20.5)	2.7 (0.4 to 8.3)	9.1 (0 to 18.1)	4.9 (0 to 12.9)
Bangladesh	20.7 (8.2 to 33.2)	26.3 (23.7 to 29.1)	0 (0 to 0)	76.5 (75.3 to 77.6)	0 (0 to 0)	0.1 (0 to 0.3)	0.1 (0 to 0.2)	14.9 (2.8 to 27)	26.3 (18.6 to 34.3)	0 (0 to 0)	29.6 (28.8 to 30.3)	0 (0 to 0)	4.9 (0 to 9.9)	2.8 (0 to 7.4)
Brazil	3.3 (0.7 to 5.9)	-15.5 (-17.4 to -13.6)	0 (0 to 0)	80 (77.5 to 82.5)	0.1 (0 to 0.1)	0.2 (0 to 0.3)	0.1 (0 to 0.3)	2.1 (0 to 4.5)	-14.3 (-19.3 to -9.7)	0.1 (0.1 to 0.1)	23.1 (22.3 to 23.8)	0.6 (0.3 to 1.2)	3.2 (0 to 6.4)	1.8 (0 to 4.9)
Central African Republic	9.7 (0 to 20.7)	12.1 (5.8 to 18.5)	0.5 (0.4 to 0.6)	15.9 (15.7 to 16.1)	2.9 (1.3 to 5.2)	0.6 (0 to 1.3)	0.4 (0 to 1)	6.7 (0 to 16.1)	11.7 (0.4 to 23.4)	0.7 (0.3 to 1.5)	24.3 (20.1 to 28.5)	3.8 (1.3 to 9.5)	10.3 (0 to 20.6)	5.5 (0 to 14.3)
China	4.4 (2 to 6.7)	-11.2 (-18.8 to -3.8)	0 (0 to 0)	43.1 (40.2 to 46)	0 (0 to 0)	0 (0 to 0)	0 (0 to 0)	2.8 (0.2 to 5.3)	-7.4 (-8.9 to -5.8)	0 (0 to 0)	16.7 (13.3 to 20.2)	0.1 (0 to 0.2)	0.5 (0 to 1)	0.3 (0 to 0.8)
Democratic Republic of Congo	11 (0.6 to 21.5)	13.9 (7.3 to 20.7)	0.1 (0.1 to 0.2)	28.4 (28 to 28.7)	0.7 (0.3 to 1.2)	0.3 (0 to 0.6)	0.2 (0 to 0.5)	7.7 (0 to 17.1)	13.8 (2.3 to 25.9)	0.2 (0.1 to 0.2)	87.3 (74.7 to 100)	0.4 (0 to 1)	6.9 (0 to 13.9)	3.8 (0 to 9.9)
Congo	8.8 (0 to 17.7)	11 (6.1 to 16.2)	0.5 (0.4 to 0.7)	14.4 (14.1 to 14.8)	3.2 (1.5 to 5.6)	0.3 (0 to 0.6)	0.2 (0 to 0.5)	6 (0 to 13.9)	10.1 (-0.9 to 21.6)	0.7 (0.3 to 1.4)	15.6 (9.9 to 21.3)	4 (1.3 to 10.5)	7.2 (0 to 14.3)	3.9 (0 to 10.4)
Ethiopia	12.2 (2.7 to 21.7)	16.7 (11.6 to 22)	0.3 (0.2 to 0.4)	30.2 (29.9 to 30.4)	1.6 (0.7 to 2.8)	0.1 (0 to 0.1)	0 (0 to 0.1)	8.5 (0 to 17.3)	17.1 (7.5 to 27.2)	0.3 (0.2 to 0.4)	47.6 (42.1 to 53)	1.4 (0.5 to 2.7)	4.7 (0 to 9.3)	2.6 (0 to 6.9)
Gabon	7.1 (0 to 15.6)	4.1 (-2.7 to 11.1)	0.3 (0.3 to 0.4)	66.3 (64.8 to 67.8)	1.1 (0.5 to 2.2)	0.2 (0 to 0.4)	0.1 (0 to 0.4)	4.8 (0 to 12.1)	3.2 (-10 to 16.7)	0.6 (0.2 to 1.3)	63.4 (31.7 to 95.1)	2.4 (0.1 to 7.7)	6.1 (0 to 12.2)	3.4 (0 to 8.7)
Indonesia	12.2 (6.8 to 17.6)	12.4 (11.2 to 13.5)	0 (0 to 0)	31 (18.5 to 43.5)	0 (0 to 0.1)	0 (0 to 0)	0 (0 to 0)	8.2 (2.2 to 14.3)	13 (7.2 to 19)	0.1 (0.1 to 0.1)	2.4 (2.3 to 2.4)	0.6 (0.3 to 1.1)	1.8 (0 to 3.6)	1.1 (0 to 2.8)
India	29.8 (22.4 to 37.1)	36.1 (34.3 to 38.1)	0 (0 to 0)	79.5 (78.3 to 80.8)	0 (0 to 0.1)	0 (0 to 0)	0 (0 to 0)	23 (13.5 to 32.5)	37 (31.4 to 42.8)	0 (0 to 0)	75.3 (64.6 to 86)	0.1 (0 to 0.2)	0.9 (0 to 1.8)	0.5 (0 to 1.5)
Kenya	9.1 (0.9 to 17.2)	10.5 (3.7 to 17.7)	0.8 (0.7 to 1)	56.7 (55.6 to 57.8)	3.2 (1.4 to 5.8)	0.1 (0 to 0.1)	0 (0 to 0.1)	6.2 (0 to 13.6)	9.5 (-3.2 to 22.7)	1.2 (0.8 to 1.7)	61.4 (59.9 to 63)	4.1 (1.5 to 8.3)	4 (0 to 8)	2.3 (0 to 6.1)
Liberia	7.6 (0.5 to 14.8)	9.4 (2.5 to 16.7)	0.1 (0.1 to 0.2)	22.3 (21.8 to 22.8)	0.8 (0.4 to 1.5)	0.3 (0 to 0.6)	0.2 (0 to 0.5)	5.1 (0 to 11.3)	10.3 (-0.3 to 21.2)	0.2 (0.1 to 0.5)	30.3 (28.8 to 31.8)	1.2 (0.3 to 3)	8.3 (0 to 16.5)	4.5 (0 to 11.8)

Lesotho	6.4 (0.2 to 12.5)	0.4 (-15.6 to 16.5)	3.1 (2.7 to 3.6)	73.9 (73.7 to 74.2)	8.3 (3.4 to 14.9)	0 (0 to 0.1)	0 (0 to 0.1)	4.6 (0 to 10.3)	-1.3 (-24.1 to 21.3)	4.6 (2.3 to 7.8)	50.1 (39.9 to 60.3)	16.2 (4.8 to 34.8)	4.8 (0 to 9.6)	2.7 (0 to 7.2)
Myanmar	15.2 (6 to 24.5)	18.6 (17.6 to 19.6)	0.1 (0.1 to 0.2)	52.8 (51.7 to 53.9)	0.4 (0.2 to 0.9)	0 (0 to 0.1)	0 (0 to 0.1)	10.5 (1.6 to 19.5)	19.4 (14.2 to 24.9)	0.1 (0.1 to 0.2)	86.8 (75.1 to 98.5)	0.2 (0 to 0.5)	2.1 (0 to 4.3)	1.2 (0 to 3.4)
Mongolia	2.9 (0 to 6.1)	-5.3 (-5.7 to -4.9)	0 (0 to 0)	100 (100 to 100)	0 (0 to 0)	0 (0 to 0)	0 (0 to 0)	1.5 (0 to 3.7)	-5.9 (-12.3 to 0.2)	0 (0 to 0)	100 (100 to 100)	0 (0 to 0)	1.9 (0 to 3.7)	1.1 (0 to 2.9)
Mozambique	4.4 (0 to 9)	3.3 (-1.7 to 8.4)	1.9 (1.6 to 2.3)	26.5 (26.4 to 26.6)	9.6 (4.7 to 16.5)	0.5 (0 to 1)	0.3 (0 to 0.8)	2.6 (0 to 6.2)	2.2 (-7.9 to 12.4)	3.8 (1.2 to 7.8)	26.6 (23.7 to 29.5)	16.8 (4.6 to 36.5)	10.1 (0 to 20.2)	5.3 (0 to 14)
Namibia	9.7 (1.6 to 17.8)	7.9 (0.8 to 15.3)	2.1 (1.8 to 2.4)	70.3 (69 to 71.5)	6.1 (2.5 to 11)	0.1 (0 to 0.3)	0.1 (0 to 0.2)	6.7 (0 to 14)	7.5 (-5.6 to 20.8)	2 (1.3 to 3)	93.3 (91.2 to 95.4)	3.7 (0.9 to 8.6)	4.5 (0 to 9)	2.6 (0 to 6.8)
Nigeria	11.4 (4.8 to 18.1)	16 (13.3 to 18.9)	0.1 (0.1 to 0.1)	58.7 (58.3 to 59.2)	0.4 (0.2 to 0.8)	0.2 (0 to 0.4)	0.1 (0 to 0.3)	7.7 (1.1 to 14.3)	15.7 (7.1 to 24.6)	0.2 (0.1 to 0.3)	65 (54.4 to 75.7)	0.7 (0.1 to 1.9)	5.8 (0 to 11.6)	3.2 (0 to 8.6)
Pakistan	22.1 (12.1 to 32.2)	27.6 (25.5 to 29.7)	0 (0 to 0)	27.6 (26.3 to 28.8)	0 (0 to 0)	0 (0 to 0.1)	0 (0 to 0)	16 (5.6 to 26.4)	27.2 (19.6 to 35.1)	0 (0 to 0.1)	2.7 (2 to 3.4)	0.1 (0 to 0.6)	2.8 (0 to 5.7)	1.6 (0 to 4.5)
Philippines	11.7 (5.2 to 18.2)	13.5 (12.5 to 14.5)	0 (0 to 0)	7.7 (7.4 to 8)	0.1 (0 to 0.1)	0 (0 to 0.1)	0 (0 to 0)	8 (1.1 to 14.9)	14.3 (8.9 to 20)	0.3 (0.1 to 0.7)	2.3 (0.5 to 4.1)	2 (0.5 to 5)	2.4 (0 to 4.8)	1.4 (0 to 3.7)
Papua New Guinea	1.6 (0 to 4.4)	-22.7 (-30.6 to -15.2)	0.1 (0 to 0.3)	38.3 (36.7 to 39.9)	0.6 (0.1 to 1.9)	0.1 (0 to 0.2)	0.1 (0 to 0.2)	1 (0 to 3.1)	-27.4 (-42 to -13.6)	0.2 (0 to 0.9)	34 (17 to 51)	1.2 (0.1 to 5.9)	3.6 (0 to 7.1)	2 (0 to 5.4)
Democratic People’s Republic of Korea	6 (0 to 13.2)	-3.7 (-9.8 to 2.3)	0 (0 to 0)	91.6 (86.1 to 97.1)	0 (0 to 0.1)	0 (0 to 0)	0 (0 to 0)	3.8 (0 to 9.7)	-3.3 (-3.4 to -3.1)	0 (0 to 0.1)	26 (18.9 to 33.1)	0.1 (0 to 0.6)	0 (0 to 0.1)	0 (0 to 0.1)
Sierra Leone	8.7 (1.4 to 16)	10.4 (4.1 to 17)	0.2 (0.1 to 0.2)	13.4 (13.3 to 13.5)	1 (0.5 to 1.7)	0.3 (0 to 0.5)	0.2 (0 to 0.4)	5.8 (0 to 12.3)	9.8 (-1.6 to 21.6)	0.4 (0.1 to 0.8)	34.6 (31.3 to 37.8)	1.9 (0.4 to 5.1)	6.4 (0 to 12.8)	3.6 (0 to 9.4)
Thailand	9.3 (3.3 to 15.4)	2.7 (-0.7 to 6.1)	0 (0 to 0.1)	100 (100 to 100)	0 (0 to 0.1)	0.1 (0 to 0.1)	0 (0 to 0.1)	6 (0.3 to 11.8)	3.4 (-0.5 to 7.6)	0.2 (0.2 to 0.3)	28.5 (27.8 to 29.3)	1.4 (0.6 to 2.6)	2 (0 to 3.9)	1.1 (0 to 3.1)
United Republic of Tanzania	7.9 (0 to 15.8)	8.5 (2.4 to 14.9)	0.6 (0.5 to 0.6)	63 (61.8 to 64.1)	1.9 (0.8 to 3.5)	0.1 (0 to 0.2)	0.1 (0 to 0.2)	5.2 (0 to 12.3)	7.5 (-4.4 to 19.7)	0.9 (0.5 to 1.3)	65.4 (61.5 to 69.4)	2.8 (1.1 to 5.5)	7.4 (0 to 14.8)	4.1 (0 to 10.6)
Uganda	6.7 (0.4 to 12.9)	7.9 (-0.7 to 17)	0.9 (0.8 to 1.1)	47.9 (47.4 to 48.5)	3.8 (1.7 to 6.8)	0.1 (0 to 0.2)	0.1 (0 to 0.2)	4.2 (0 to 9.4)	6.3 (-8.2 to 21)	1.2 (0.7 to 2)	65.2 (60.4 to 70)	3.9 (1.1 to 9.2)	7.2 (0 to 14.4)	4 (0 to 10.4)
Viet Nam	16.9 (8.9 to 25)	21.9 (19.2 to 24.5)	0 (0 to 0)	100 (100 to 100)	0 (0 to 0)	0 (0 to 0)	0 (0 to 0)	11.3 (3.1 to 19.5)	22.6 (18.9 to 26.6)	0.1 (0 to 0.2)	19 (5.5 to 32.6)	0.5 (0.1 to 1.7)	2.2 (0 to 4.4)	1.3 (0 to 3.4)
South Africa	5.6 (1.2 to 9.9)	-7.9 (-14.5 to -1.5)	2.2 (1.9 to 2.5)	55.4 (53.9 to 56.8)	8.1 (3.7 to 14)	0.1 (0 to 0.1)	0 (0 to 0.1)	3.1 (0 to 6.7)	-16.4 (-35.1 to 1.6)	3.9 (1.9 to 6.6)	40.6 (35.3 to 45.9)	15.4 (4.8 to 32.2)	3.3 (0 to 6.7)	1.9 (0 to 5)
Zambia	7.4 (0 to 15.1)	7.1 (0.7 to 13.7)	1.4 (1.2 to 1.6)	46.4 (46.3 to 46.5)	5.9 (2.7 to 10.3)	0.2 (0 to 0.4)	0.1 (0 to 0.3)	4.9 (0 to 11.8)	6.3 (-5.9 to 18.8)	2.3 (1.2 to 3.7)	60.5 (59.8 to 61.2)	7.6 (2.6 to 16)	7.7 (0 to 15.4)	4.2 (0 to 10.9)
